# Assessing the genetic diversity and characterizing genomic regions conferring Tan Spot resistance in cultivated rye

**DOI:** 10.1371/journal.pone.0214519

**Published:** 2019-03-28

**Authors:** Jagdeep Singh Sidhu, Sai Mukund Ramakrishnan, Shaukat Ali, Amy Bernardo, Guihua Bai, Sidrat Abdullah, Girma Ayana, Sunish K. Sehgal

**Affiliations:** 1 Department of Agronomy, Horticulture & Plant Science, South Dakota State University, Brookings, SD, United States of America; 2 Department of Plant Pathology, Kansas State University, Manhattan, KS, United States of America; 3 USDA-ARS, Hard Winter Wheat Genetics Research Unit, Manhattan, KS, United States of America; National Cheng Kung University, TAIWAN

## Abstract

Rye (*Secale cereale* L.) is known for its wide adaptation due to its ability to tolerate harsh environments in semiarid areas. To assess the diversity in rye we genotyped a panel of 178 geographically diverse accessions of four *Secale* sp. from U.S. National Small Grains Collection using 4,037 high-quality SNPs (single nucleotide polymorphisms) developed by genotyping-by-sequencing (GBS). PCA and STRUCTURE analysis revealed three major clusters that separate *S*. *cereale L*. from *S*. *strictum* and *S*. *sylvestre*, however, genetic clusters did not correlate with geographic origins and growth habit (spring/winter). The panel was evaluated for response to *Pyrenophora tritici-repentis* race 5 (*PTR* race 5) and nearly 59% accessions showed resistance or moderate resistance. Genome-wide association study (GWAS) was performed on *S*. *cereale* subsp. *cereale* using the 4,037 high-quality SNPs. Two QTLs (*QTs*.*sdsu-5R* and *QTs*.*sdsu-2R*) on chromosomes 5R and 2R were identified conferring resistance to *PTR* race 5 (*p* < 0.001) that explained 13.1% and 11.6% of the phenotypic variation, respectively. Comparative analysis showed a high degree of synteny between rye and wheat with known rearrangements as expected. *QTs*.*sdsu-2R* was mapped in the genomic region corresponding to wheat chromosome group 2 and *QTs*.*sdsu-5R* was mapped to a small terminal region on chromosome 4BL. Based on the genetic diversity, a set of 32 accessions was identified to represents more than 99% of the allelic diversity with polymorphic information content (PIC) of 0.25. This set can be utilized for genetic characterization of useful traits and genetic improvement of rye, triticale, and wheat.

## Introduction

Rye (*Secale cereale* L.) belongs to the Triticeae tribe of the family Poaceae [1] and is believed to share a common ancestor with wheat (*Triticum aestivum* L.) and barley (*Hordeum vulgare* L.) [2]. Most of the species of genus *Secale* originated in the Middle East, particular in Turkey [3]. Later along with the dissemination of wheat and barley to Europe and the Western Mediterranean regions, rye first came as a weed to these places. From the weedy species of rye, farmers consciously or unconsciously selected variants with a non-brittle rachis and larger seeds [3], which became current *Secale cereale*, the only cultivated species of rye. Due to its resilience, rye first adapted as a secondary crop in the areas with harsh environments (cold and heat stress), where other staple crops like wheat and barley were not able to survive [3]. Eventually, seeing its versatility, farmers started cultivating rye. Now, it is grown in Canada and northern parts of the United States, Russia, Japan, Australia, and South Africa [4].

In general, the genus *Secale* is classified into four species: *S*. *cereale—*an annual allogamous species, *S*. *sylvestre and vavilovii—*an annual autogamous species and *S*. *strictum—*a perennial wild-type allogamous species [5]. Around the globe, rye is cultivated mainly for food, feed, and pasture; and also, as a cover crop or green manure crop. Rye-based products are a rich source of nutritionally essential compounds such as minerals (Zn, Fe, and P), β-glucan (1.3–2.7%), starch, and dietary fibers [6,7]. In Europe, rye grain provides a substantial portion of the human (as bread) and animal diet. In North America, rye is preferably grown as a cover crop or pasture, and its grain is used as livestock feed and for alcohol distillation. In dry lands of southern Australia, it is grounded to prevent wind erosion. Furthermore, due to its sturdiness, it is also considered as a good pioneer crop to restore the fertility of waste lands [4].

Triticale (X *Triticosecale* Wittmack), a cross between durum wheat (AABB) and rye (RR) further signifies the stress tolerance ability of rye by producing relatively higher biomass and grain yield over the other cereals in dry and cold environments [8]. Through chromosome substitutions or translocations, important genes from rye have been exploited for the improvement of other cereals especially wheat [9]. One of the important examples signifying the pest resistance of rye is 1BL.1RS translocation in wheat. Rye chromosome arm 1RS carries savior genes conferring resistance to stem rust (*Sr31*), leaf rust (*Lr26*), powdery mildew (*Pm8*), and yellow rust (*Yr9*) [10–12]. Likewise, there are many other wheat-rye translocations harboring stress-resistance genes that aided in increasing the grain yield and the adaptation potential of bread wheat [13–16]. Rye offers great potential for wheat improvement and should be further explored [17].

Assessing the genetic diversity in rye can help broaden the genetic base of rye, enhance accessibility to the important genes and improve management of gene bank resources.[18]. Genetic diversity analysis involves the comparison of the accessions for their dissimilarities at the molecular level to determine the variation present in a set of accessions. Genetically manipulating a large collection of accessions could be costly and laborious, therefore extracting a core set that represents the genetic diversity of the entire set is a promising methodology [19–21]. A core set of germplasm can make the available germplasm resources to be more systematically and easily utilized in breeding programs by eliminating redundancy [19].

Among the diploid species of Poaceae family, rye has the largest genome (~7.9 Gbps) [22] with about 90% of repetitive sequences [23]. Due to the genome complexity and its regional cultivation, the rye genome has not been extensively studied. Several studies on rye genetic diversity have been conducted using different marker systems including SSR (Simple sequence repeats) [5,24–28], AFLP (Amplified Fragment Length Polymorphism) [29], DArT (Diversity Arrays Technology) [21,30], and recently SNPs (Single nucleotide polymorphism) [31]. Majority of these studies either used a limited number of markers covering a small portion of a genome or may have ascertainment bias. GBS (genotyping-by-sequencing) provides an opportunity for simultaneous SNP discovery across a genome and analysis of the genetic diversity, population structure and evolution processes of any species. Recently a draft sequence of the rye genome has been released, which will facilitate SNP marker development and the molecular characterization of economically important traits in rye.

Identification of linked molecular markers to genes of interest may help understand the molecular mechanisms of gene actions and facilitate marker-assisted selection of important traits in breeding and wide hybridization. Several genetic linkage maps have been developed in rye [32–35]. A number of genes/QTLs have been mapped in rye including plant height [36,37], spike length [37], number of spikelets per spike [37], benzoxazinoid content, rust resistance, α-amylase activity, and preharvest sprouting resistance [38]. However, linkage mapping has limitations in capturing the available genetic diversity and has a lower capacity to detect polygenic traits. With the availability of large-scale SNP data, genome-wide association studies (GWAS) provide an opportunity to characterize and map genes for important traits. GWAS is based on linkage disequilibrium (LD), and tightly linked genes theoretically have high LD which is maintained over generations [39]. GWAS has been used to characterize several economically important traits such as yield, disease, pest resistance, and abiotic stress tolerance in many crop species including rice [40–44], maize [45–51], barley [52–58], and wheat [59,60,69–71,61–68].

Rye has been reported to carry several important resistance genes and QTLs [9,10,12,13]. However, GWAS has not been widely used to identify genes/QTLs and linked markers in rye. In this study, we used GWAS approach to characterize the rye tan spot, an economically important disease caused by a necrotrophic fungus *Pyrenophora tritici-repentis* (*PTR*) and has caused up to 49% yield loss under favorable conditions [72]. Rye is known to be infected with tan spot and but QTLs for resistance to *PTR* race 1 and *PTR* race 5 has not been reported. Identification of additional genes/QTLs for tan spot resistance in rye could facilitate the development of tan spot resistant wheat, rye, and triticale varieties.

The goals of the current study were to assess the genetic diversity in cultivated rye and develop a smaller diverse representative set to facilitate genetic improvement in rye, triticale and wheat through the development of new germplasm and to demostrate the potential of GWAS in rye by identifying QTL(s) conferring resistance to tan spot *PTR* race 5.

## Materials and methods

### Plant materials

**Fig 1:**
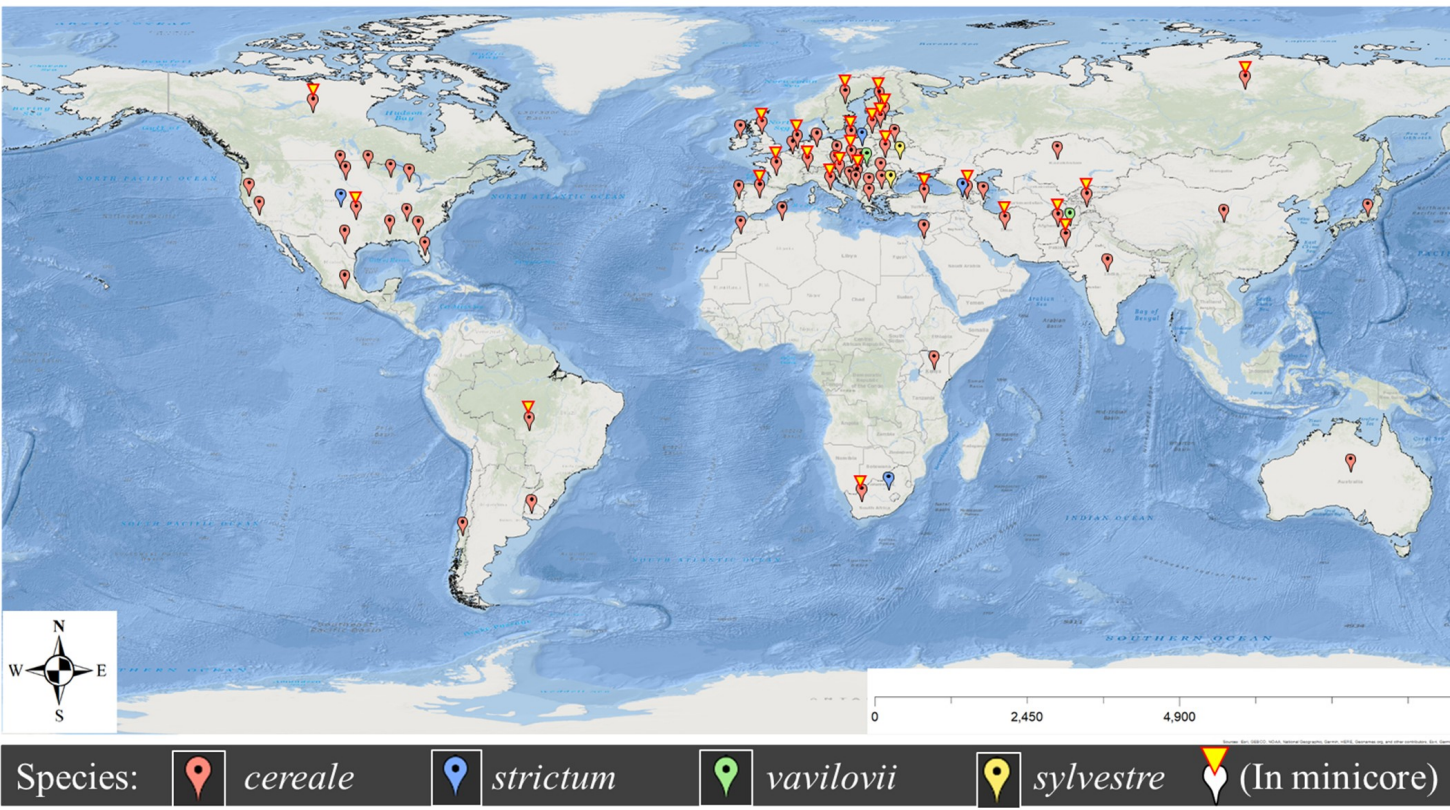
Geographic diversity of the panel of 178 accessions and the smaller diverse representative set of 32 accessions of *Secale* sp. Color code: red, blue, green, and yellow map pins, correspond to *Secale cereale* subsp., *S*. *strictum* subsp., *S*. *vavilovii*, and *S*. *sylvestre*, respectively. Overlaid yellow triangles refer to accessions in smaller diverse representative set, which was selected from 160 cultivated rye (*S*. *cereale* subsp. *cereale*) accessions.

A panel of 178 geographically diverse (70 countries) accessions of *Secale* sp. was selected from the USDA National Small Grains Collection (NSGC). The majority of the accessions are from the Middle East (primary center of origin) and Europe (secondary center of origin) (Fig 1). The panel includes 160 cultivated rye (*S*. *cereale* subsp. *cereale*), nine wild *S*. *cereale* subsp., five *S*. *strictum*, and two each of *S*. *sylvestre and S*. *vavilovii* (S1 and S2 Tables). Only *S*. *cereale* subsp. *cereale* accessions were used as an association panel and to extract a smaller diverse representative set.

### Genotyping and SNP discovery

Young leaf tissues were collected from three-week-old plants of each accession for DNA isolation using the hexadecyltrimethylammonium bromide (CTAB) method [73]. The DNA was quantified and normalized to 20ng/μl. GBS was performed following the double-restriction enzyme digestion protocol for library construction and Ion Proton system sequencing [74]. Briefly, 10μl of the normalized DNA from each accession was double-digested with *PstI* and *MspI* restriction enzymes (New England BioLabs, Inc., Ipswich, MA, USA) and then ligated to two adapters with barcodes [74–76]. After the adapters were ligated, the samples were pooled together for PCR amplification and subsequently size-selected for 250-300bp amplicons using the E-gel system (Thermo Fisher Scientific, Waltham, MA, USA). Libraries (80 pM) were prepared and sequenced in an Ion Proton system using Ion PI Hi-Q Chef and Sequencing 200 kits with two Ion P1v3 chips (Thermo Fisher Scientific, Waltham, MA, USA). SNPs were called using a reference-based SNP calling pipeline of TASSEL 5 [77]. A rye custom reference genome was obtained from the Plant Genome and Systems Biology (PGSB) website (http://pgsb.helmholtz-muenchen.de/plant/rye/gz/download/) [78] and used as a reference to locate the SNPs within the rye genome.

### Population structure and genetic diversity

Basic genetic diversity indices: polymorphic information content (PIC) and Shanon’s diversity index (I-index) were calculated for each SNP using the formula:

$$PIC=1-(p^{2}{+q}^{2})\;and\;I=-\Sigma p_{i}{log}_{2}p_{i}$$

Where, p and q correspond to the major and minor allele frequency, respectively [79], and p_i_ is the allele frequency of the i^th^ allele at a particular locus [80]. Percentage dissimilarity based principal coordinate analysis (PCA) among and between the species was performed using a R-package prcomp [81]. For comparison among accessions, a pairwise genetic dissimilarity (GD) matrix was computed using a R-package ape [82]. GD was employed for hierarchical clustering and a neighbor-joining (NJ) tree was constructed using R-package fastcluster [83]. Finally, the tree was pictographically developed using an online tool, Tree of life (iTOL) [84].

Population structure among all *Secale* sp. accessions was analyzed using STRUCTURE software [85]. DeltaK, the estimated likelihood [LnP (D)], method described by Evano et al. [86] was used to determine the optimum number of clusters. This method is based on a change in the log probability of the data in question, moving from successive K values. The number of clusters (K) with the highest value of DeltaK was selected. Accessions with > 60% ancestry from one particular cluster were grouped into corresponding population e.g. accessions in P1 population carries > 60% ancestry from cluster 1 (C1). Accessions with < 60% ancestry from any single cluster were declared admixture of two clusters with which they share > 20% ancestry. For example, accessions in the cluster P12 shared ancestries of cluster 1 (C1) and cluster 2 (C2).

### Selection of the smaller diverse representative set of rye

To identify a diverse representative set to represent the diversity of 160 accessions of *S*. *cereale* subsp. *cereale*, the association panel was first classified into distance-based clusters. From the clusters containing less than 10 accessions, a single accession that represents the corresponding cluster was selected as a member of the smaller set. If a cluster had larger than 10 accessions, it was further sub-divided such that each sub-cluster had less than 10 accessions. Then, the most representative accession was selected from each of the sub-clusters to ensure the PIC value of the smaller diverse represenative set was maximized.

### Evaluation of reaction to *Pyrenophora tritici-repentis* (*PTR*) race 5

Nine seeds of each genotype were planted in three cones (3.8 cm in diameter and 20 cm in length) with three seeds per cone as one replication. Wheat genotypes 6B662 and Salamouni were used as susceptible and resistant checks, respectively. Plants were grown in a greenhouse at temperatures ranging from 20 to 23°C with 16 h photoperiod till inoculation. At the second leaf stage, plants were inoculated with *PTR* race 5 using a spore suspension of 2,500 spores/ml. Inoculated plants were moved to a mist chamber (18°C) for 24 h and then grown for 7 d in a greenhouse at 21°C with 16 h photoperiod. Disease lesions were rated in a 1–5 scale [87] with 1 as resistant, 2 as moderately resistant, 3 moderately susceptible, and 4 and 5 as susceptible at the 7^th^-day post-inoculation (S1 Fig). The experiment was repeated once under the same growing conditions. The average reaction score of each accession from the two experiments was used for GWAS analysis (S2 Table).

### GWAS analysis

Genome-wide association for resistance to *PTR* race 5 was conducted using R package GAPIT (Genome Association and Prediction Integrated Tool) [88]. Three linear models, namely GLM (generalized linear model), MLM (mixed linear model), and CMLM (compressed mixed linear model), were evaluated. GLM is based on the least square fixed effects [88], whereas, MLM includes both fixed and random effects. The fixed effects in our case were the SNP effect and population structure (Q), and the random effect was for relatedness among the individuals (kinship). MLM model is mathematically denoted as:

y=Xβ+Zu+e

Where, “y” is the vector of phenotypic values (categorical values in our case), “β” is the vector containing fixed effects namely SNP effects and Q, “u” is the vector of the random effects, which in our case is random genetic effects from multiple background QTL that were not controlled by markers (kinship). “X” and “Z” are known incidence matrixes for corresponding vectors. Kinship matrix was calculated using GAPIT’s kinship algorithm–VanRaden method [89] and Q matrix was obtained using principal component analysis [90]. CMLM is an extension of MLM, in which the individuals were clustered into groups using the group-based kinship matrix rather than individual based [91]. We primarily focused on MLM. The markers with a p-value < 1.0 ×10^−3^ or log_10_ (p-value) > 3 were considered to be significantly associated with the trait. We performed 5-fold jackknife validation to confirm the accuracy of significant marker-trait association (MTA) [92]. Briefly, the entire panel of 160 accessions was divided into five random sub-groups and four of them were used for association analysis, each time leaving one random group out. Results were also compared with the results from TASSEL 5.0 [77].

### Comparative analysis of rye and wheat

To study the synteny between wheat and rye genomes, specifically for genomic regions conferring resistance against *PTR* race 5 in rye, a comparative analysis was conducted between the two genomes. Flanking sequence (150 base pair) of each 4,037 SNPs including the candidate SNPs identified from MTAs were retrieved from the rye reference genome. A 300bp long sequence for each SNP was compared with IWGSC wheat genome assembly RefSeq v1.0 [93] using BLASTn (*E*-value cut off of 1E^-50^ with an identity higher than 75%.) [94]. The comparative analysis results were visualized using a Perl based software Circos [95].

## Results

### Genotype-by-sequencing for genome-wide SNP discovery

We obtained a total of 178,598,329 sequence reads from a GBS library prepared from 178 rye accessions. Using the reference-based pipeline, 27,882 SNPs with 80% or fewer missing genotypes were identified (NCBI SRA accession: PRJNA512245). On an average, each chromosome has 4,000 SNPs (Table 1), with maximum (5,505) on chromosome 5R and minimum (2,536) on the chromosome 6R (S4 Table). To keep only the most informative SNPs, we removed 7,113 indel markers. A total of 4,037 high-quality SNPs that have less than 20% missing genotypes, less than 10% heterozygotes and above 5% MAF (minimum allele frequency) were used for further analysis. The final selected set of SNPs showed a similar trend of distribution on the seven chromosomes with an average of 577 SNPs per chromosome, maximum of 734 SNPs on chromosome 5R and a minimum of 358 SNPs on chromosome 6R (Table 1).

**Table 1:** Number and chromosome location of SNPs discovered by genotyping-by-sequencing of 178 rye accessions.

Chromosome	Total SNPs	Filtered SNPs*
1R	3,468	504
2R	3,914	600
3R	3,916	605
4R	5,505	685
5R	4,774	734
6R	2,536	358
7R	3,892	551
**Total**	**28,005**	**4,037**

*Number of SNPs with 20% or less missing genotypes, heterozygotes less than 10% and MAF >5%

### Genetic variability in the rye germplasm collection

The average PIC value for the 4,037 SNPs present in 160 *S*. *cereale* subsp. *cereale* accessions was 0.26, ranging from 0.09 to 0.5. About 38% SNPs had PIC values ranging from 0.1 to 0.2, 26% SNPs had PIC values of 0.2 to 0.3, 19% had PIC values of 0.3 to 0.4, and only 14% had PIC values 0.4 to 0.5. The PIC values for SNPs on each chromosome followed the similar pattern of distribution. Average PIC values were 0.25 for 6R, 7R, and 4R; 0.26 for 2R; and 0.27 for 1R, 3R, and 5R (S2 Fig). The average I-index for 4,037 SNPs in 160 *S*. *cereale* subsp. *cereale* accessions was 0.48. Among 18 accessions of wild species, average PIC value and I-index were 0.25 and 0.57, respectively.

**Fig 2:**
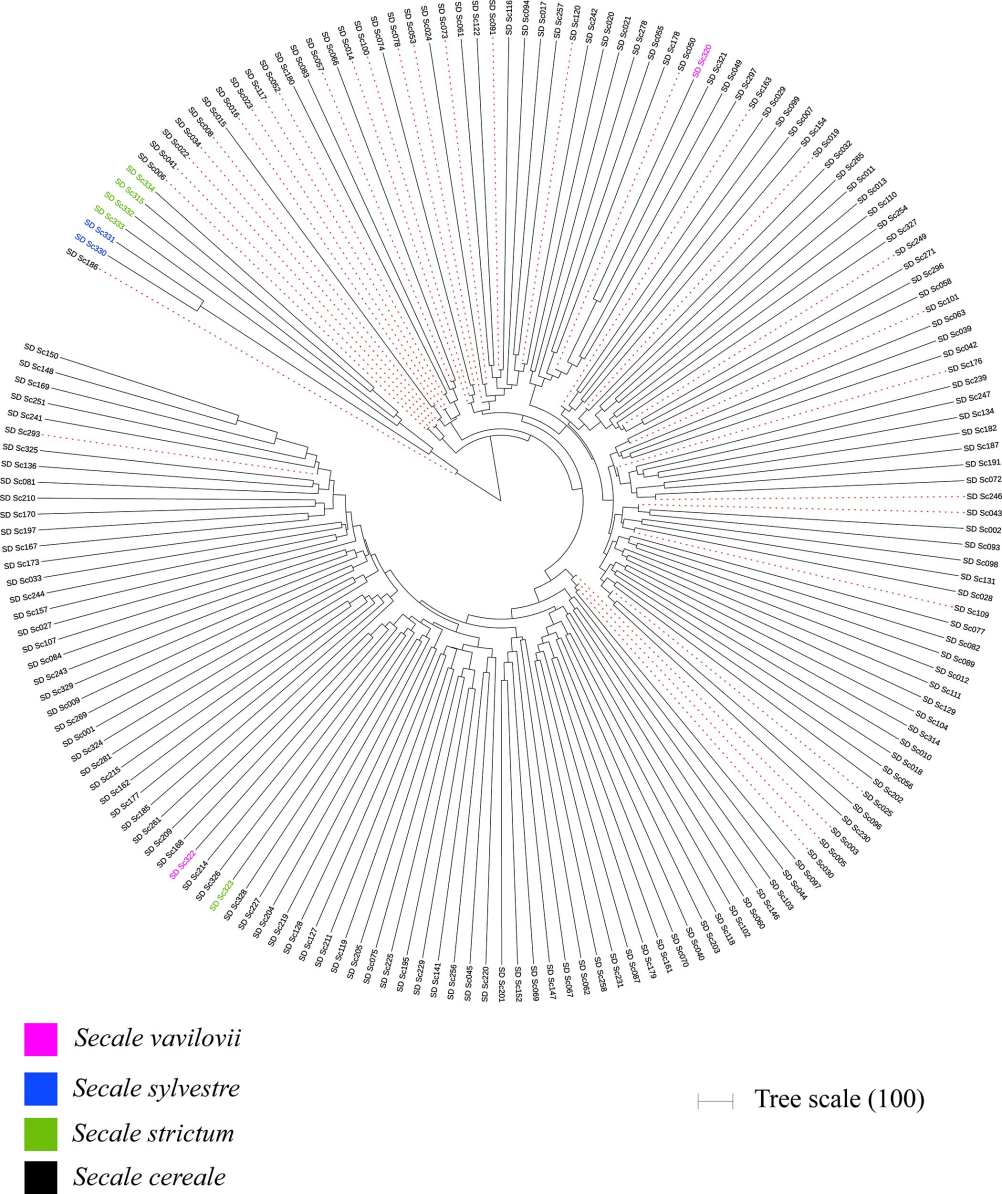
Pairwise dissimilarity-based neighbor-joining tree. Smaller diverse representative set (red-dash clades) represents all the major clusters of *Secale cereale* subsp. *cereale*. *S*. *strictum* (green) and *S*. *sylvestre* (blue) and *S*. *vavilovii* (pink) are also shown.

The average percentage dissimilarity (GD) among the entire panel of *S*. *cereale* subsp. *cereale* was 0.48, ranging from 0.26 to 0.63. The lowest GD (0.26) was found between the accessions SD_Sc150 and SD_Sc148, and the highest GD (0.63) was found between SD_Sc195 and SD_Sc186. Average GD for individual chromosomes ranged from 0.46 to 0.49 (S3 Fig). The average GD among wild species (18 accessions) was 0.51, ranging from 0.15 to 0.66. Among the wild species, SD_Sc330 (*S*. *sylvestre*) and SD_Sc322 (*S*. *vavilovii*) were the most distant accessions, and SD_Sc330 (*S*. *sylvestre*) and SD_Sc331 (*S*. *sylvestre*) were the most similar accessions with 0.66 and 0.15 GD, respectively. GD matrix-based farthest Neighbor-joining phylogenetic tree (Fig 2 & S5 Fig) accurately clustered the three species, *S*. *cereale*, *S*. *strictum*, and *S*. *sylvestre*, into three different clusters, except for SD_Sc323, the only spring type accession of *S*. *strictum*, which falls in a cluster of *S*. *cereale*. On the contrary, *S*. *vavilovii* clades were found scattered within a cluster of *S*. *cereale*. The spring type accessions of *S*. *vavilovii* (SD_Sc322) was found in the same cluster as spring type accession of *S*. *strictum*. *S*. *sylvestre* and *S*. *strictum* were found to be closer to each other as compared to *S*. *cereale*.

## Population structure and principal component analysis (PCA)

**Fig 3:**
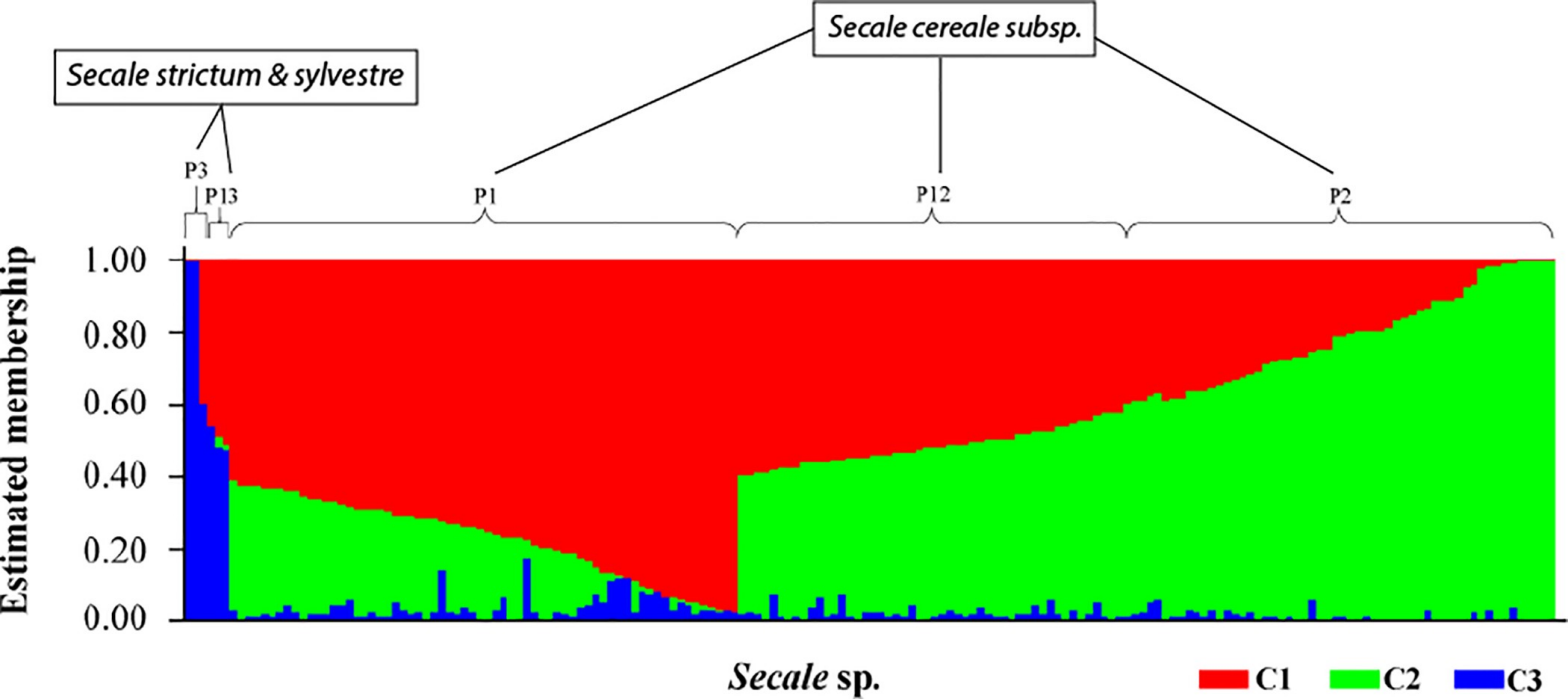
Structure analysis on (K = 3) 178 *Secale* sp. accessions. Y-axis represents the estimated membership of individuals from populations and X-axis represents 178 *Secale* sp. accessions. Accessions are ordered according to the population ancestry.

Bayesian clustering (STRUCTURE) analysis was performed on the 178 *Secale* sp. accessions and the estimated likelihood [LnP (D)] was found to be greatest at K = 3, suggesting three major populations that explain most of the genetic variation (Fig 3). Among all the accessions, 67% (120) belongs to one of the three unique populations (P1, P2, and P3) with > 60% ancestry contributed by anyone corresponding cluster. The populations P1, P2, and P3 contain 66, 51, and three accessions, respectively. Other 32% (58) of the accessions have admixtures in their ancestries (> 20% contribution by two clusters), in which 55 accessions in P12 have ancestries of both C1 and C2, whereas, P13 has only three accessions having ancestries of both C1 and C3. No accession shared significant ancestry (> 20%) between C2 and C3. Accessions of *S*. *cereale* subsp. were mainly found in P1, P2, and P12, whereas, P3 and P13 consisted of *S*. *strictum* and *S*. *sylvestre*.

**Fig 4:**
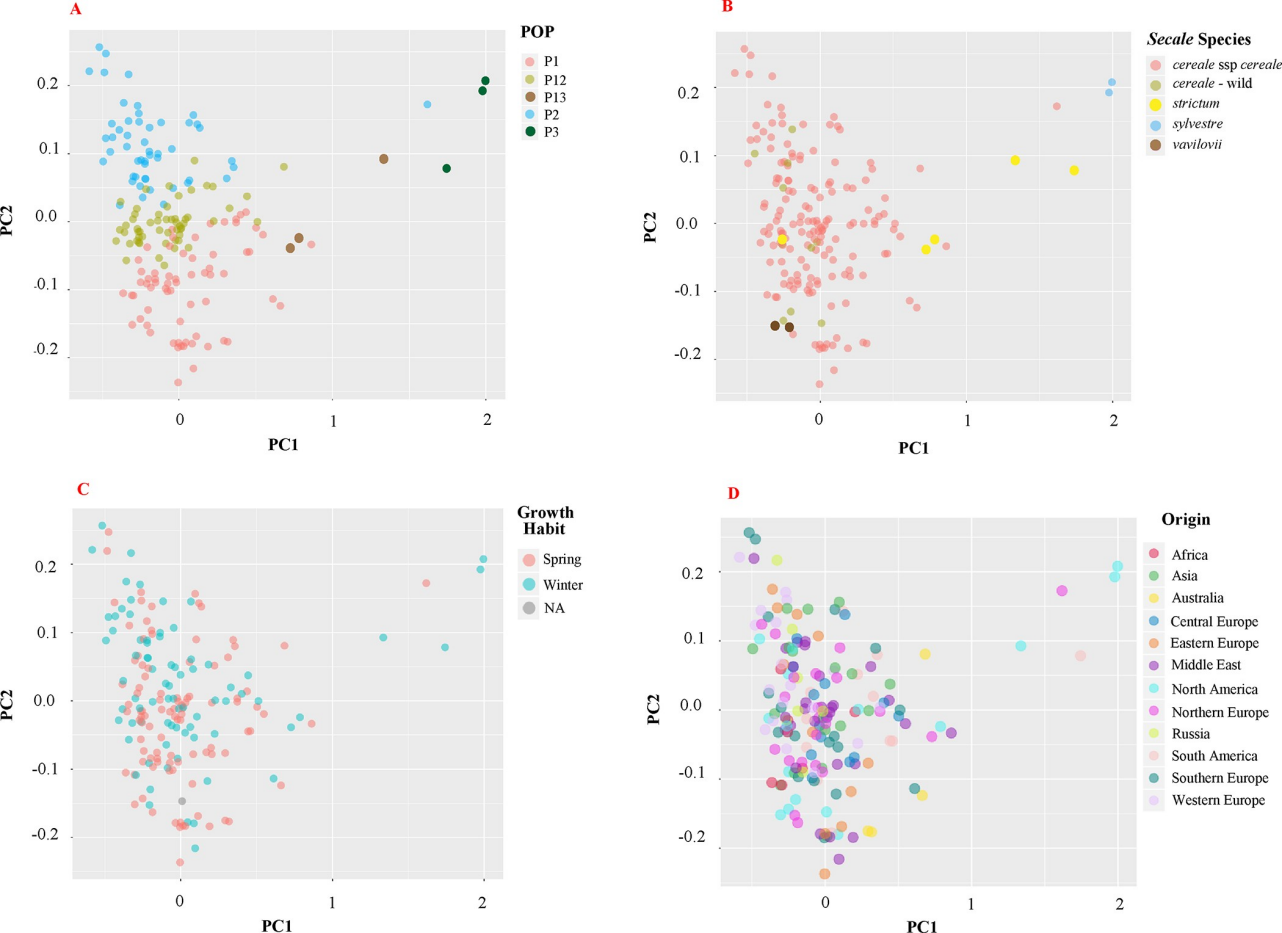
Principal component analysis (PCA) on 178 *Secale* sp. accessions to show the relationship of genetic clusters with population structure. (A), *Secale* species investigated (B), spring or winter type habit (C), and the geographic origin (D).

GD-based PCA results were basically consistent with the model-based population structure (Fig 4A). First and second PCA explained 40% and 3% of the genetic diversity, respectively. Three main populations (P1, P2, and P3) are clearly separated. The two admixtures (P12 and P23) lie between the corresponding populations with which they share ancestry. P3 mostly consist of wild species of *S*. *strictum* and *S*. *sylvestre* and is separated from rest of the evaluated accessions (Fig 4B). One accession of *S*. *strictum* was found in the population of *S*. *cereale* subsp. Interestingly, this accession is the only spring type accession of *S*. *strictum*. We also found *S*. *vavilovii* accessions mixed with the *S*. *cereale*. Correlation between genetic clusters and growth habit (spring vs winter, Fig 4C) or geographic origin (Fig 4D) was not observed. Geographic regions were divided according to Bolibok-Bragoszewska et al. (2014), in which Europe is divided into five regions: east, west, south, north, and central; and other countries are combined into corresponding broad geographic regions such as Middle East, Asia, South America, North America, Australia, and Russia [21].

### Selection of the smaller diverse representative set of rye

A total of 32 accessions were extracted as the smaller diverse representative set from 160 accessions of *S*. *cereale* subsp. *cereale* (PIC = 0.2518). The smaller diverse representative set contains only 20% of the accessions evaluated, but it covered 99% of the allelic diversity in the entire set. The smaller diverse representative set includes all the main clusters in NJ tree, with a minimum of one accession from each cluster (Fig 2). Accessions within a cluster are closer to each other as compared to accessions between clusters. The smaller diverse representative also captured a large portion of the geographic diversity (27 countries) of the entire collection (70 countries) of *S*. *cereale* subsp. *cereale* to represent the major geographic regions (Fig 1). The average PIC value and I-index of the smaller diverse representative set are not significantly (p < 0.01) different from the original whole set (Table 2). Average GD is significantly (p < 0.01) higher among accessions within the smaller diverse representative as compared to the original whole set (Table 2).

**Table 2:** Comparison of the diversity indices between smaller diverse representative set and geographically diverse set of 160 accessions of *Secale cereale* subsp. *cereale*.

	Size	Average PIC	Average I-index†	Average GD‡
Geographically diverse Set	160	0.26	0.60	0.48
Smaller representative set	32	0.25	0.59	0.51
T-test (p-value)		0.02	0.11	1.90e^-90^*

†Shannon’s diversity index

‡Pairwise genetic dissimilarity

*Significant at α <0.01.

### Reactions of rye accessions to *Pyrenophora tritici-repentis* race 5

A set of 178 accessions of *S*. *cereale* were evaluated for resistance to tan spot (*PTR* race 5), however, only 160 accessions of *S*. *cereale* subsp. *cereale* were used for GWAS analysis. We observed a range of responses to *PTR* race 5 inoculations with 31.8% (51) resistant accessions (R—category 1), 26.9% (43) moderately resistant accessions (MR—category 2), 24.4% (39) moderately susceptible (MS—category 3), and 16.8% (27) of susceptible accessions (S—categories 4 and 5) (S4 Fig). As expected, the resistant check (Salamouni) showed resistant (Score—1) response and the susceptible check (6B662) produced chlorosis reaction with a score of 4 to 5. All these results were consistent in both experiments.

### Marker-trait association (MTA) for tan spot (*PTR* race 5) resistance in rye

**Fig 5:**
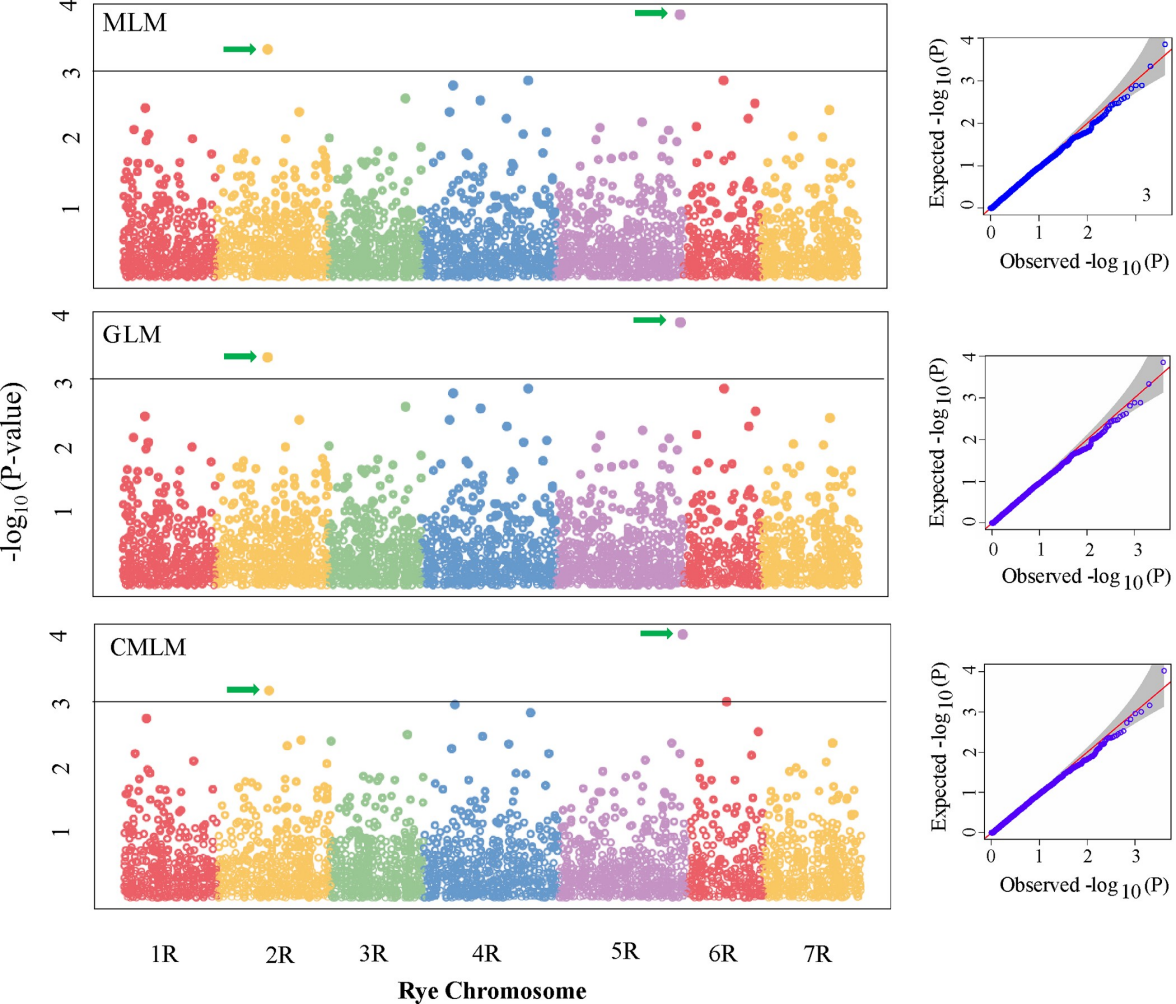
Genome-wide association scan for tan spot (*PTR* race 5) resistance in rye. Three different model-based Manhattan plots represent–log_10_ (p-value) for SNPs distributed across all seven chromosomes of rye. Y-axis:–log_10_ (p-value) and x-axis: rye chromosomes. The dashed line stands as a threshold for significant markers with–log_10_ (p-value) of > 3 which correspond to a p-value <1 × 10^−3^. The arrows pointed two significant SNPs. On the right side of each model, Quantile-Quantile (QQ) plots represent expected null distribution of p-values vs observed p-values.

Among the tested linear models, we focused on MLM, because of obvious kinship and population structure detected for the population. Two genomic regions were associated with tan spot (*PTR* race 5) resistance in rye, with one region on chromosome 2R (*QTs*.*sdsu-2R*) and the other on 5R (*QTs*.*sdsu-5R*). The SNPs “*S5R_16433036”* (p = 1.4 × 10^−4^) on chromosome 5R and “*S2R_6856816”* (p = 4.5 × 10^−4^) on chromosome 2R explained 13.1% and 11.6% of the phenotypic variation, respectively (Fig 5). The two SNPs associated with tan spot resistance identified with the MLM algorithm were consistent with the results derived from GLM, and CMLM algorithms (Fig 5). The candidate SNPs were also validated using 5K jackknife approach, and both were consistent in the five repetitions with a p-value ≤ 1.0 ×10^−3^.

### Comparative analysis of rye and wheat

**Fig 6:**
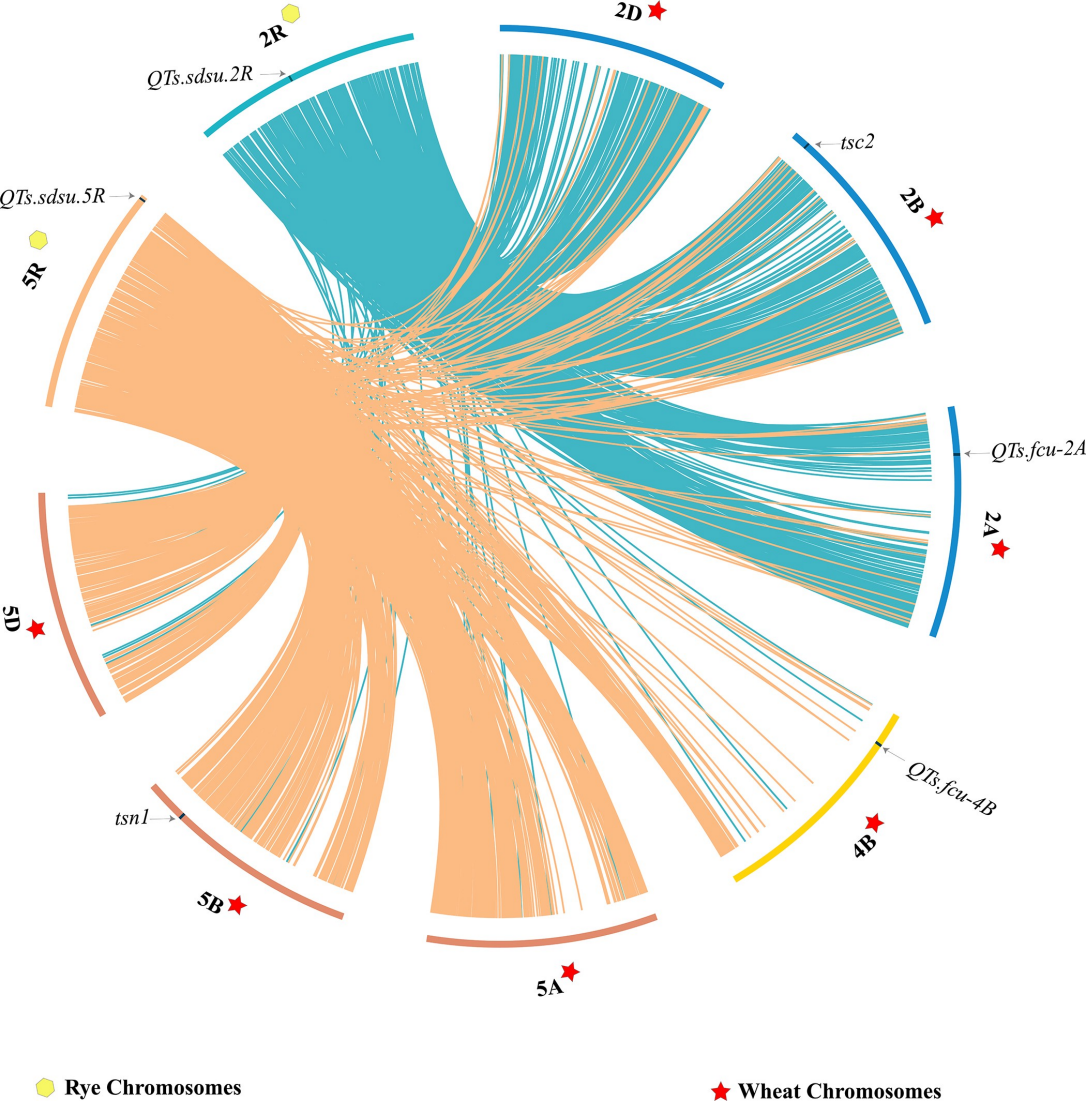
Circular genome data visualization of synteny between wheat homoeologous groups (Group 2 – 2A, 2B, & 2D; Group 5 – 5A, 5B, & 5D; chromosome 4B) and rye chromosomes (2R and 5R) harboring tan spot (*PTR* race 5) resistance QTLs discovered in our study. Each chromosome clockwise–short arm to long arm. *QTs*.*sdsu-5R* and *QTs*.*sdsu-*2R are shown on their corresponding rye chromosomes. *QTs*.*sdsu-*2R has a hit on wheat chromosome group 2 which harbors tan spot insensitivity gene—tsc 2 (2B) and tan spot resistance QTL (*QTs*.*fcu-2A*) (2A). *QTs*.*sdsu-5R* hits a small segment on wheat chromosome arm 4BL which harbors no tan spot related QTL, however, *QTs*.*fcu-4B* has been reported on the chromosome arm 4BS in wheat.

Syntenic analysis was conducted to compare the marker sequences of the identified QTL regions between wheat and rye. The SNPs flanking *QTS*.*sdsu-2R* had a hit on wheat group 2 which has a tan spot (*PTR* race 5) insensitivity gene (*tsc2*) on chromosome 2B and a QTL (*QTs*.*fcu-2A*) on chromosome 2A (Fig 6). *S5R_16433036* in the *QTs*.*sdsu-5R* region showed a hit on a small segment on the terminal (tip) region of the long arm of wheat chromosome 4B although 5R is mainly syntenic with group 5 of wheat (Fig 6). No tan spot related QTL has been reported on the long arm of 4B, however, *QTs*.*fcu-4B* has been reported on the chromosome arm 4BS. Comparative analysis between rye genome (7 chromosomes) and their corresponding wheat homeologous groups (group 1–7) showed a general trend of synteny between the two genomes (S6 Fig). Majority of chromosomes 1R, 2R, 3R, and 5R are syntenic to wheat corresponding homeologous groups (1, 2, 3, and 5), respectively. However, blocks of rye chromosome 4R showed synteny with wheat groups 4, 6, and 7. Chromosome 6R is syntenic to wheat groups 6 and 3, because of fewer markers were found on 6R, the synteny with 6R was not very clear. Chromosome 7R shared syntenic blocks with wheat groups 5, 4, and 7.

## Discussion

### Rye genome coverage by GBS-SNPs

Assessing the genetic diversity in germplasm resources using DNA markers can help in better exploitation of germplasm for crop improvement. In rye, several diversity studies have been conducted using a limited number of DNA markers ranging from few dozens of markers [5,24,27] to hundreds [28,29,31], probably due to laborious genotyping methods and technological limitations. Furthermore, chromosomal positions of these markers were not reported. To address this issue of anonymous marker positions and low genome coverage, one study has reported using 1,054 DArT markers to cover seven chromosomes of rye [21]. In the present study, we employed more than 4000 GBS-SNPs that provided much better coverage of the genome than in all the previous studies.

To our knowledge, this is the first report of double enzyme digestion-based GBS used in rye. GBS is a next-generation-sequencing based method that generates a large number of SNPs and shows advantages for high diversity species like rye. We found more than 4,000 polymorphic SNPs distributed evenly across all the seven chromosomes of rye except chromosome 6R, which has lower number (358) than the average number of SNPs per chromosome (576) (Table 1). With GBS, the number of SNPs discovered in a genomic region is directly correlated to the level of its genetic diversity [96], This suggests that chromosome 6R is likely less diverse than the other rye chromosomes, which is in line with several previous studies [1,2,21,30].

### Genetic diversity in rye

Among 178 accessions investigated in this study, 160 are cultivated accessions of *S*. *cereale* subsp. *cereale* that cover most of the geographic diversity of the cultivated rye. In this rye collection, the average PIC value for all the SNPs is 0.26, which is lower than several previously reported values [21, 96–98). High PIC values from previous studies might be due to the use of carefully and deliberately selected markers [97], or due to the use of multi-allelic markers as for multi-allelic markers PIC values range from 0 to 1 but 0 to 0.5 for bi-allelic markers such as SNPs. However, lower PIC of SNPs can be overweighed by their enormous number and genome-wide distribution, thus giving a similar or even better estimation of genetic diversity. The similar PIC values across chromosomes in this study indicate that polymorphic SNPs were evenly distributed on all the seven rye chromosomes.

Average GD value among *S*. *cereale* subsp. *cereale* was 0.48 with a range from 0.26 to 0.63, which is comparable with other studies in rye [5,21,98]. Accessions SD_Sc195 and SD_Sc186 have highest dissimilarity index of 0.63. These accessions being most diverse may be of future interest for exploiting heterosis. Average GD among wild species (0.51) was higher as compared to cultivated species, which is in accordance with the expectation that wild species conserve larger diversity [21]. Therefore, wild species can further be exploited to infuse diversity into cultivated germplasm. SD_Sc330 (*S*. *sylvestre*) and SD_Sc322 (*S*. *vavilovii*) were the most diverse accessions.

Results among all three clustering approaches (Bayesian clustering, PCA, and Neighbor-Joining clustering) were consistent. Bayesian clustering predicted three populations: P1, P2, and P3. P1 and P2 both consisted of *S*. *cereale* subsp. and *S*. *vavilovii* accessions; P3 consisted of *S*. *sylvestre* and *S*. *strictum* accessions, reported in previous studies as well [30,98]. These clusters were also seen with PCA. Genome composition of *S*. *sylvestre* was 100% from the P3 population, whereas, *S*. *strictum* had about 10 to 20% from P1. Shared ancestry among some of the accessions of *S*. *strictum* and *S*. *cereale* subsp. group (P1) supports the hypothesis that *S*. *strictum* is the potential ancestor of *S*. *cereale* [3,99–101]. Unlike other wild *Secale* sp., *S*. *vavilovii* accessions were found in the clusters of *S*. *cereale* subsp, which is in accordance with previous reports [30,98], suggesting its classification may need to be revisited. Wild species of *S*. *cereale* cannot be separated out of the clusters of the *S*. *cereale* subsp. *cereale* in our study similar to previous studies [21], suggesting an active gene transfer among these species.

After reviewing the geographic origin of accessions in different genetic clusters, we did not find any correlation between genetic cluster and geographic origin. This may be due to the sharing of the common genetic background among the accessions being analyzed in each study as it is also reported by other independent studies [5,21,27,29]. It has been reported that vernalization requirement can lead to population divergence in rye [98], triticale [102], and wheat [103]. We examined growth habit (winter vs spring) of the genetic clusters and did not see a substantial association between clusters and growth habit or vernalization requirement.

### Use of the smaller diverse set in representing the geograhically diverse set of cultivated rye

Mini core sets have been established for number of crops including wheat [104,105], rice [106], maize [107], soybean [108], and rye [109]. Adding one more collection to that list, we identified a smaller diverse representative set of 32 accessions representing genetic (99% alleles) and geographic diversity (all major regions) of 160 accessions of *S*. *cereale* subsp. *cereale*. The PIC value and I-index of the smaller diverse representative is comparable to those of the whole set while average GD is significantly higher than the whole set. Thus, the smaller diverse representative carries similar genetic information as the whole set, which can be easily and efficiently used for rye gene mining and transferring important genes into X. *Triticosecale* and wheat. Also, our smaller diverse representative set contains only one-fifth of the accessions from the original set but retain 99% of the allelic diversity, thus demonstrating that germplasm banks can significantly reduce the germplasm conservation cost without the loss of genetic information if they genotype entire available germplasm and develop core diversity sets.

### Identification of putative genomic regions conferring tan spot (*PTR* race 5) resistance

Rye is known for its resilience to the abiotic and biotic stress tolerance [109] and it has contributed a number of important genes into wheat germplasm [9,11,12,110]. Previously, we reported that rye might carry resistance genes to tan spot [111], but QTLs have not been defined. Using a GWAS panel of 160 accessions of *S*. *cereale* subsp. *cereale* we identified two putative loci conferring resistance to *PTR* race 5. The two SNPs (*S2R_6856816”* on chromosome 2 and “*S5R_16433036”* on chromosome 5) collectively explained 24.7% of the phenotypic variation.

Comparative analysis between rye and wheat revealed that the significant marker linked to tan spot resistance on chromosome 2R is homologous to chromosome group 2 in wheat. Interestingly, chromosome group 2 of wheat carries many tan spot resistance or insensitivity related genes including *tsc2* (*PTR* race 5) on chromosome 2B, and *QTs*.*fcu-2A* (*PTR* race 5) and another resistance QTL (*PTR* race 1) on chromosome 2A [112–114]. However, the incomplete genome assembly of rye and no information on the sensitivity of rye accessions to Ptr ToxB, we don't know if the QTL on 2R is syntenic to the wheat gene *tsc2*. The QTL *QTs*.*sdsu-5R* had a most significant hit on wheat chromosome 4B. Though most of the chromosome 5R of rye is syntenic to chromosome group 5 of wheat, a small 5R segment hits a region on the long arm of chromosome 4B which includes our candidate SNP. Though no tan spot resistance QTL is reported so far on the terminal segment of chromosome 4BL (region hit by *QTs*.*sdsu-5R*), however, a QTL, *QTs*.*fcu-4B*, has been reported on chromosome arm 4BS [115]. Thus, *QTs*.*sdsu-5R* may harbor novel genes for *PTR* race 5 resistance. The QTLs identified in our study can be easily transferred using linked SNPs into wheat and triticale for improving tan spot resistance in these crops. Using similar approach, genes/QTLs controlling agronomic traits and tolerance to biotic and abiotic stress can be mapped in rye and transferred to triticale and wheat.

## Conclusions

Our study reports the rye genetic diversity analysis using GBS and identifies a smaller diverse representative set of 32 accessions that retains ~99% of the allelic diversity. This set of 32 accessions can be used as parents for rye, triticale, and wheat improvement. Genetic clustering was neither linked with geographic origins and nor with growth habit, suggesting individuals shared a common genetic background due to germplasm exchange and no major genomic changes happened due to vernalization requirements. Further, using GWAS we identified two genomic regions conferring resistance to tan spot (*PTR* race 5) in rye and the tightly linked SNPs *S5R_16433036* (*QTs*.*sdsu-5R*) and *S2R_6856816* (*QTs*.*sdsu-2R*) can be used for marker-assisted selection of the tan spot resistance genes.

## Supporting information

S1 Fig Different response reactions to tan spot (*Pyrenophora tritici-repentis* race 5). 1 –Resistant wheat Salamouni (check), 2 –Resistant rye, 3 –Moderately susceptible rye, 4 –Susceptible rye.
(TIF)

S2 Fig Distribution of PIC values of SNPs from 160 *Secale cereale* subsp. *cereale* accessions on each chromosome of rye. X-axis: PIC value and Y-axis rye chromosomes. Violin plots show the density distribution of SNPs for the chromosome corresponding PIC values. Box plots represent first and third quartiles. Horizontal white bars are corresponding median PIC value and yellow dot stands for average PIC value.
(TIF)

S3 Fig Distribution of pairwise dissimilarity values among *Secale cereale* subsp. *cereale* for the total number of markers corresponding to each chromosome of rye. X-axis: GD–pairwise genetic dissimilarity–percentage and Y-axis rye chromosomes. Violin plots show the density distribution of pairwise dissimilarities values. Box plots represent first and third quartiles. Horizontal white bars are corresponding median pairwise dissimilarity and yellow dot stands for average pairwise dissimilarity corresponding to each chromosome.
(TIF)

S4 Fig Distribution of *Secale cereale* subsp. *cereale* accessions among response categories to tan spot (*PTR* race 5). 1 = resistant, 2 = moderately resistant, 3 = moderately susceptible and 4 & 5 = susceptible.
(TIF)

S5 Fig Pairwise dissimilarity-based unrooted neighbor-joining tree. Smaller diverse representative set (red-dash clades) represents all the major clusters of *Secale cereale* subsp. *cereale*. *S*. *strictum* (green) and *S*. *sylvestre* (blue) and *S*. *vavilovii* (pink) are also shown.
(TIF)

S6 Fig Synteny between wheat and rye genomes showing the locations of tan spot resistance and insensitivity genes/QTL locations. Black bars on rye chromosomes denotes SNP density (SNPs/10Mb) on the rye chromosomes. *QTs*.*sdsu-5R* and *QTs*.*sdsu-*2R are presented adjacent to their corresponding rye chromosomes. Red italics denotes the mapped tan spot insensitivity genes (*tsn1*, *tsc1*, and *tsc2*) and resistance genes (*tsr2*, *tsr3*, *tsr4*, and *tsr5*) adjacent to their corresponding wheat chromosomes. Wheat is an allohexaploid species (2*n* = 6*x* = 42) with three (A, B, and D) homoeologous chromosome sets and rye is a diploid species (2*n* = 2*x =* 14).
(TIF)

S1 Table Number of accessions of each *Secale* sp. in the diversity set of 178 accessions. These accessions represent 70 different countries around the globe.
(DOCX)

S2 Table A detailed description of the *Secale* sp. accessions used in this study. For each accession, its country of origin, corresponding PI number, species, population, and response to tan spot (*PTR* race 5) is given. Populations are based on structure results.
(DOCX)

S3 Table Nucleotide sequence flanking the SNPs associated with tan spot (*PTR* race 5) resistance in a collection of *Secale cereale* accessions. (DOCX)

S4 Table Genotyping by sequencing (GBS) with 4,037 SNPs in 178 accessions of *Secale* sp. Information on 4,037 GBS SNPs and genotype of 178 accessions at each of these SNP locations in HapMap format.
(TXT)
