# Supplementary figures and images for: Assessing the genetic diversity and characterizing genomic regions conferring Tan Spot resistance in cultivated rye
Source: PLoS One. 2019 Mar 28;14(3):e0214519. doi: 10.1371/journal.pone.0214519 (PMC6438500; doi:10.1371/journal.pone.0214519)

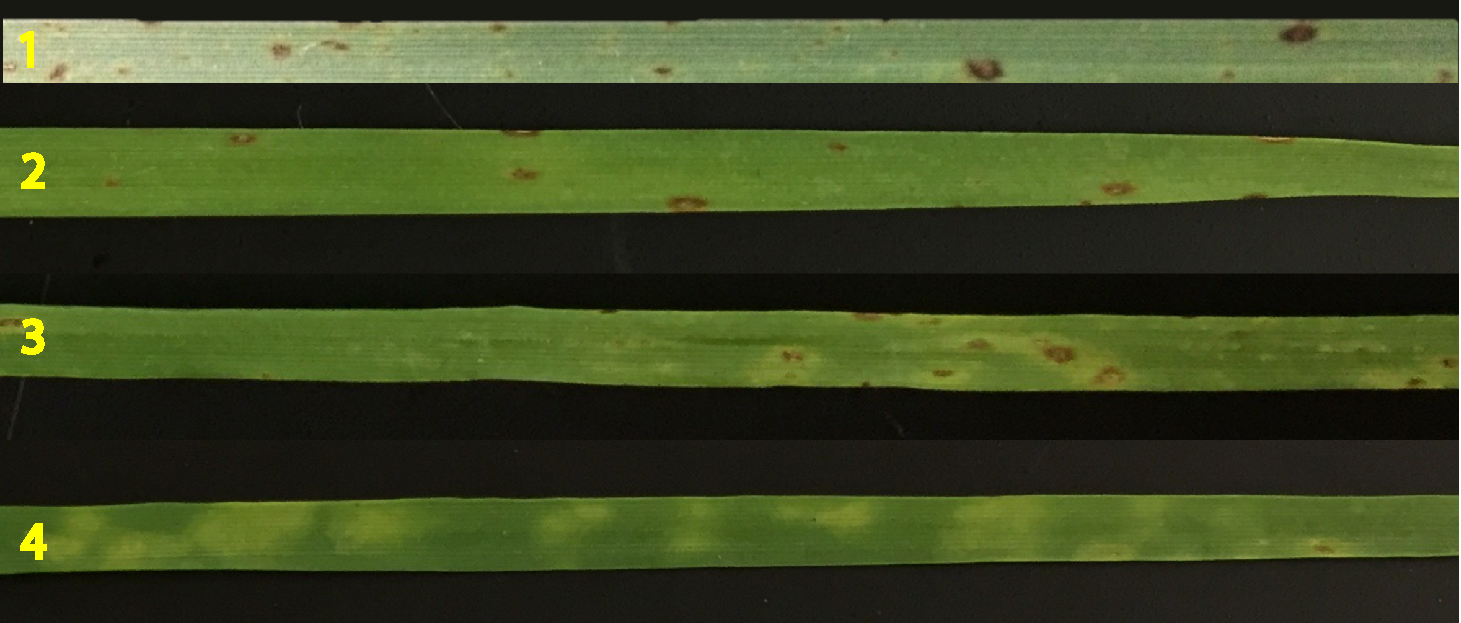

Supplement: S1 Fig — 1 –Resistant wheat Salamouni (check), 2 –Resistant rye, 3 –Moderately susceptible rye, 4 –Susceptible rye. (TIF) [file pone.0214519.s001.tif]

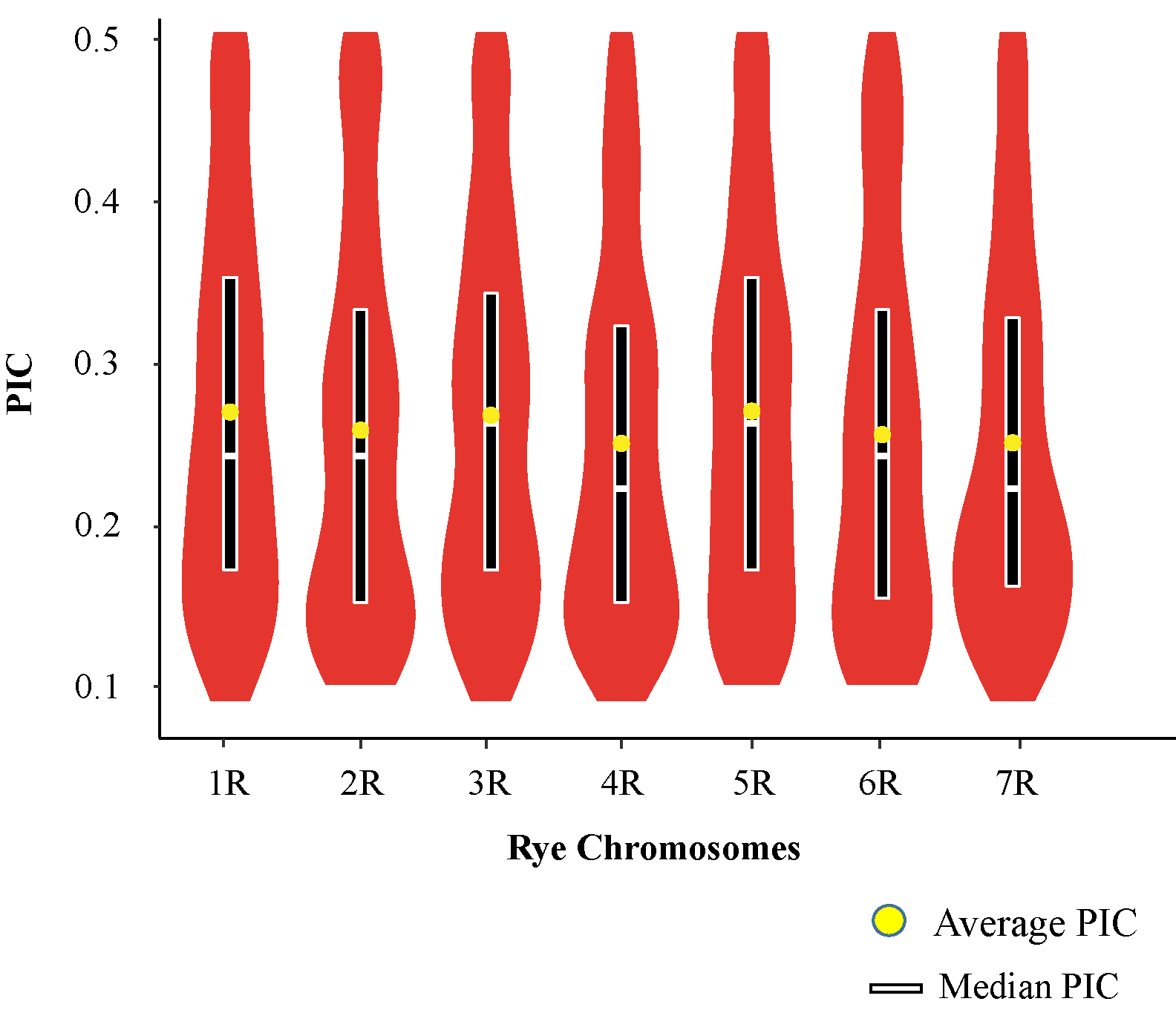

Supplement: S2 Fig — X-axis: PIC value and Y-axis rye chromosomes. Violin plots show the density distribution of SNPs for the chromosome corresponding PIC values. Box plots represent first and third quartiles. Horizontal white bars are corresponding median PIC value and yellow dot stands for average PIC value. (TIF) [file pone.0214519.s002.tif]

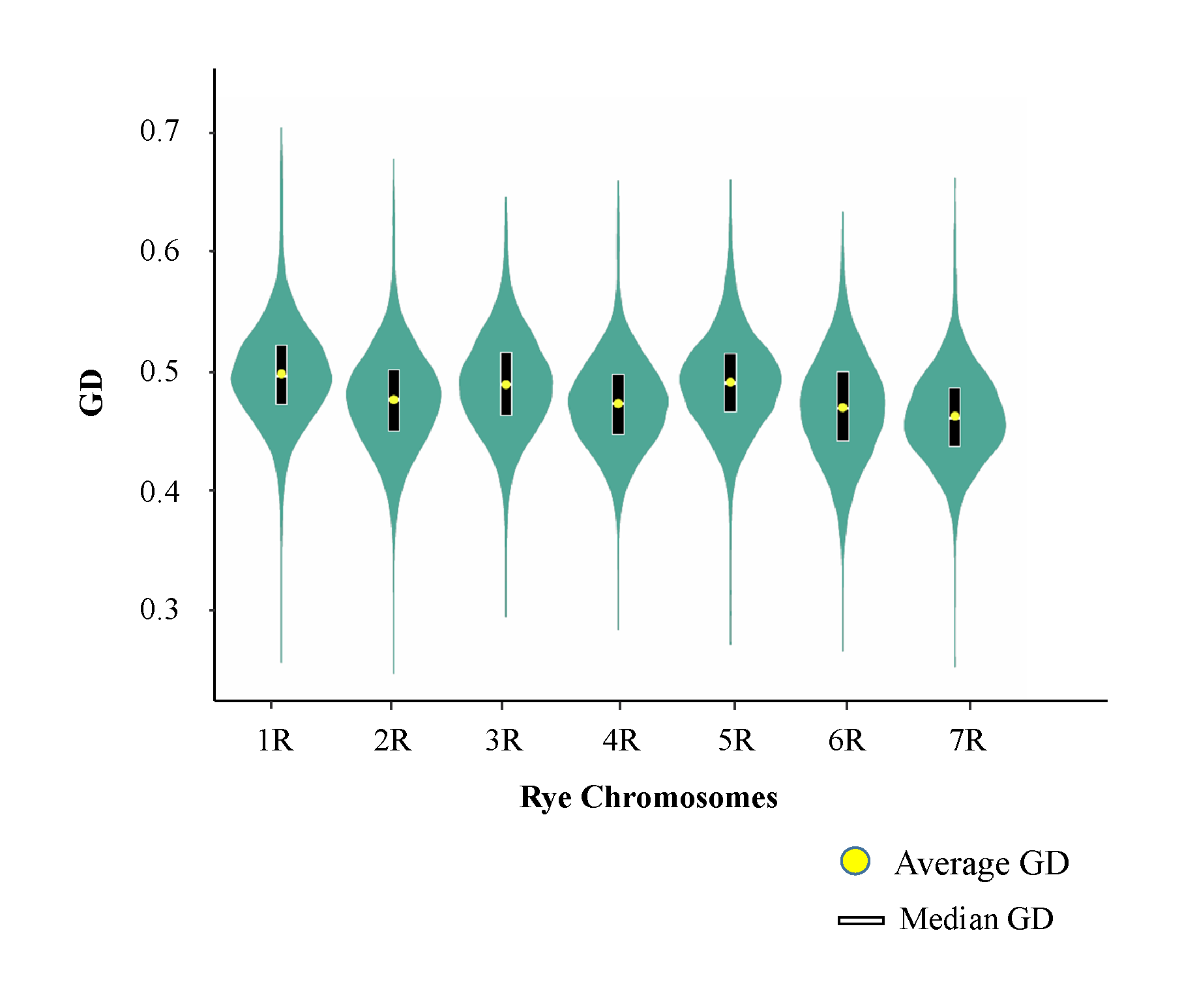

Supplement: S3 Fig — X-axis: GD–pairwise genetic dissimilarity–percentage and Y-axis rye chromosomes. Violin plots show the density distribution of pairwise dissimilarities values. Box plots represent first and third quartiles. Horizontal white bars are corresponding median pairwise dissimilarity and yellow dot stands for average pairwise dissimilarity corresponding to each chromosome. (TIF) [file pone.0214519.s003.tif]

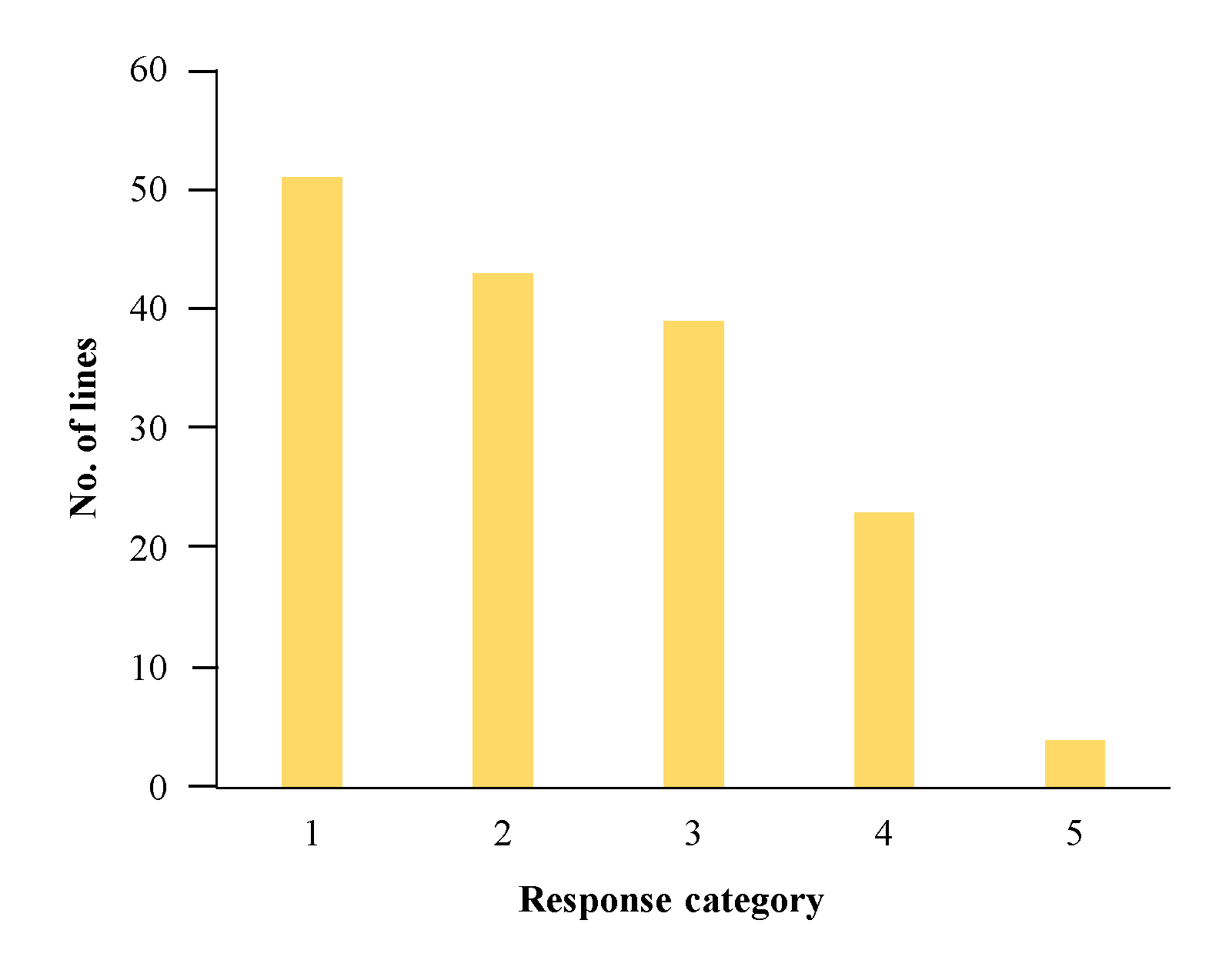

Supplement: S4 Fig — 1 = resistant, 2 = moderately resistant, 3 = moderately susceptible and 4 & 5 = susceptible. (TIF) [file pone.0214519.s004.tif]

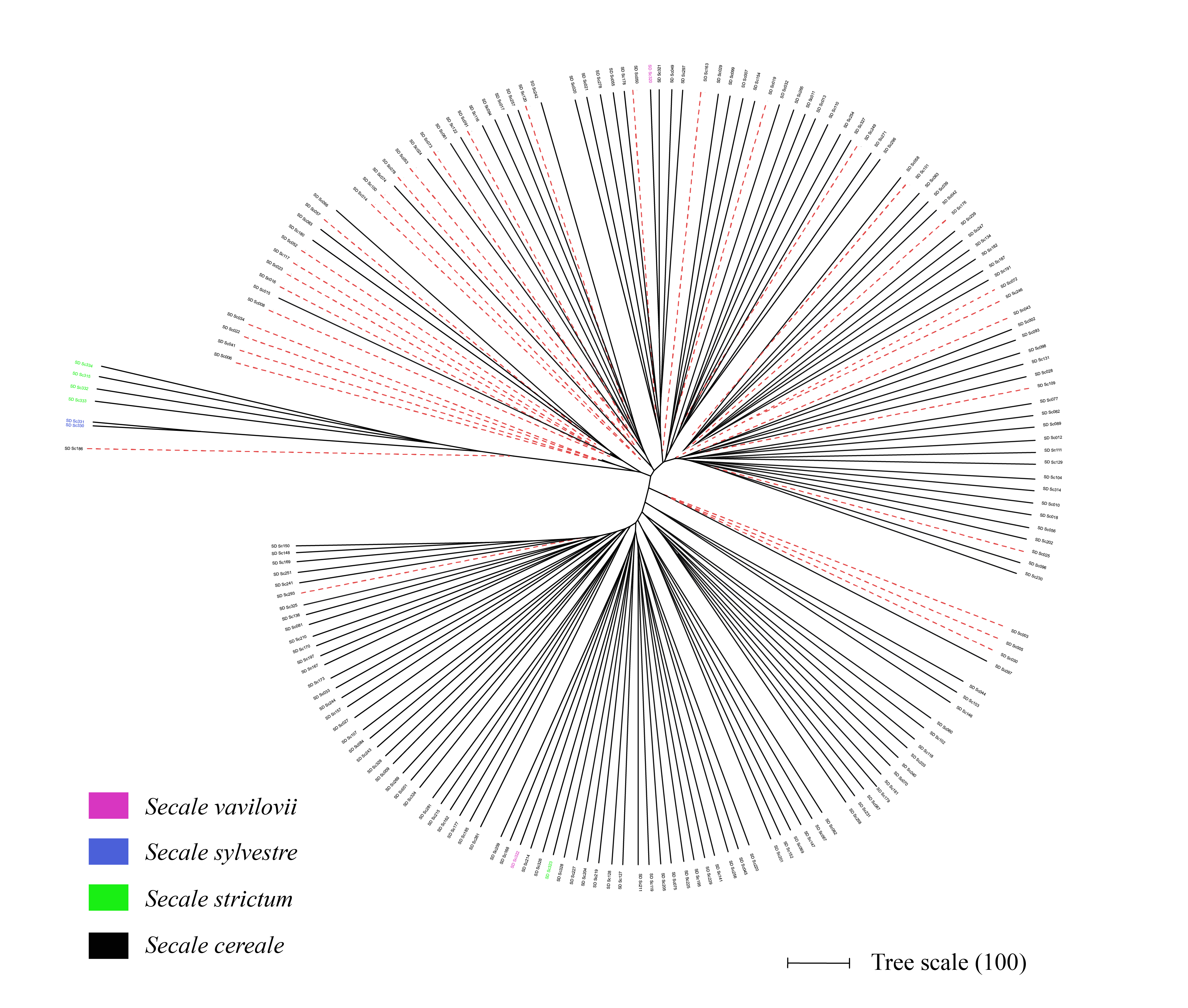

Supplement: S5 Fig — Smaller diverse representative set (red-dash clades) represents all the major clusters of Secale cereale subsp. cereale. S. strictum (green) and S. sylvestre (blue) and S. vavilovii (pink) are also shown. (TIF) [file pone.0214519.s005.tif]

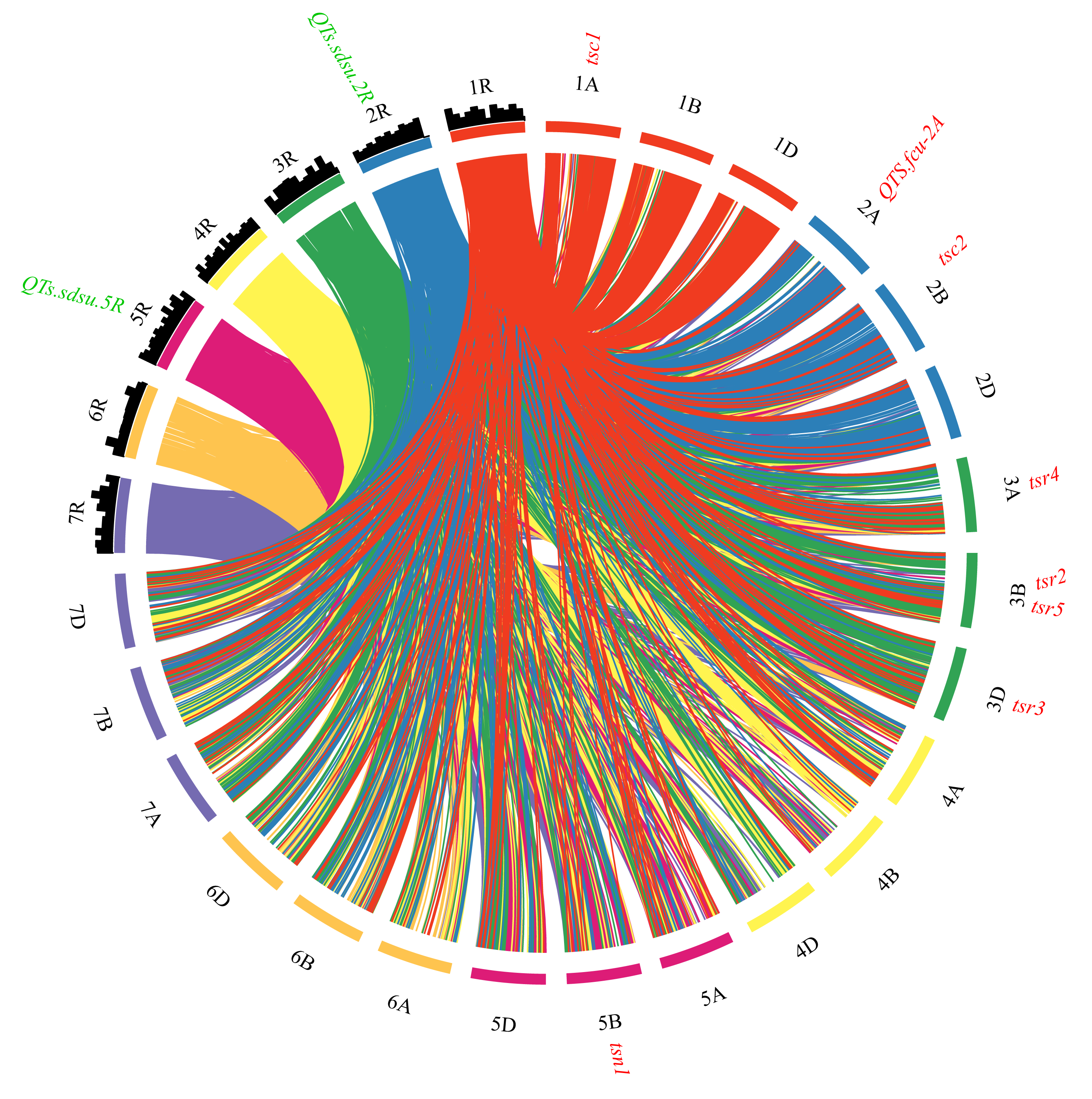

Supplement: S6 Fig — Black bars on rye chromosomes denotes SNP density (SNPs/10Mb) on the rye chromosomes. QTs.sdsu-5R and QTs.sdsu-2R are presented adjacent to their corresponding rye chromosomes. Red italics denotes the mapped tan spot insensitivity genes (tsn1, tsc1, and tsc2) and resistance genes (tsr2, tsr3, tsr4, and tsr5) adjacent to their corresponding wheat chromosomes. Wheat is an allohexaploid species (2n = 6x = 42) with three (A, B, and D) homoeologous chromosome sets and rye is a diploid species (2n = 2x = 14). (TIF) [file pone.0214519.s006.tif]
