# Supplementary material for: Assessing the genetic diversity and characterizing genomic regions conferring Tan Spot resistance in cultivated rye
Source: PLoS One. 2019 Mar 28;14(3):e0214519. doi: 10.1371/journal.pone.0214519 (PMC6438500; doi:10.1371/journal.pone.0214519)
Supplement: S1 Table — These accessions represent 70 different countries around the globe. (DOCX) [file pone.0214519.s007.docx]

**S1 Table.** Number of accessions of each *Secale* subsp. in the diversity panel of 178 accessions collected from 70 countries.

| **Sr.no.** | **Genera** | **Species** | **Subspecies** | **No. of lines** |
| --- | --- | --- | --- | --- |
| **1** | *Secale* | *cereale* | *cereale* | 160 |
| **2** | *Secale* | *cereale* | *tetraploidum* | 1 |
| **3** | *Secale* | *cereale* | *afghanicum* | 1 |
| **4** | *Secale* | *cereale* | *dighoricum* | 1 |
| **5** | *Secale* | *cereale* | *segetale* | 2 |
| **6** | *Secale* | *cereale* | *Unranked rigidum* | 1 |
| **7** | *Secale* | *cereale* | *ancestrale* | 3 |
| **8** | *Secale* | *vavilovi* | *-* | 2 |
| **9** | *Secale* | *strictum* | *anatolicum* | 1 |
| **10** | *Secale* | *strictum* | *strictum* | 1 |
| **11** | *Secale* | *strictum* | *siliatoglume* | 1 |
| **12** | *Secale* | *strictum* | *kupriganovi* | 1 |
| **13** | *Secale* | *strictum* | *africanum* | 1 |
| **14** | *Secale* | *sylvestre* | *-* | 2 |
