# Supplementary material for: Assessing the genetic diversity and characterizing genomic regions conferring Tan Spot resistance in cultivated rye
Source: PLoS One. 2019 Mar 28;14(3):e0214519. doi: 10.1371/journal.pone.0214519 (PMC6438500; doi:10.1371/journal.pone.0214519)
Supplement: S2 Table — For each accession, its country of origin, corresponding PI number, species, population, and response to tan spot (PTR race 5) is given. Populations are based on structure results. (DOCX) [file pone.0214519.s008.docx]

**S2 Table.** A detailed description of the *Secale* ­sp. accessions used in this study. For each accession, its country of origin, corresponding PI number, species, population, and response to tan spot (*PTR* race 5) is given. Populations are based on structure results.

| SD_code | Country | PI No. | *Genera* | *species* | subsp. | Population | Ptr (race5) |
| --- | --- | --- | --- | --- | --- | --- | --- |
| SD_Sc001 | Sweden | Cise 1 | *Secale* | *cereale* | *cereale* | P2 | 1.83 |
| SD_Sc002 | Sweden | Cise 20 | *Secale* | *cereale* | *cereale* | P12 | 3.83 |
| SD_Sc003 | United States | Cise 28 | *Secale* | *cereale* | *cereale* | P1 | 1.00 |
| SD_Sc005 | United States | Cise 38 | *Secale* | *cereale* | *cereale* | P1 | 1.50 |
| SD_Sc006 | Australia | Cise 79 | *Secale* | *cereale* | *cereale* | P1 | 2.00 |
| SD_Sc007 | France | Cise 84 | *Secale* | *cereale* | *cereale* | P12 | 1.00 |
| SD_Sc008 | Bosnia and Herzegovina | PI 349919 | *Secale* | *cereale* | *cereale* | P1 | 3.00 |
| SD_Sc009 | Ireland | Cise 106 | *Secale* | *cereale* | *cereale* | P12 | 1.17 |
| SD_Sc011 | Japan | Cise 108 | *Secale* | *cereale* | *cereale* | P12 | 1.00 |
| SD_Sc012 | Japan | Cise 109 | *Secale* | *cereale* | *cereale* | P12 | 3.00 |
| SD_Sc013 | Korea, South | Cise 110 | *Secale* | *cereale* | *cereale* | P1 | 3.00 |
| SD_Sc014 | United States | Cise 174 | *Secale* | *cereale* | *cereale* | P1 | 4.00 |
| SD_Sc015 | United States | Cise 176 | *Secale* | *cereale* | *cereale* | P2 | 3.00 |
| SD_Sc016 | Canada | Cise 183 | *Secale* | *cereale* | *cereale* | P12 | 3.00 |
| SD_Sc017 | United States | Cise 521 | *Secale* | *cereale* | *cereale* | P2 | 3.00 |
| SD_Sc018 | Israel | PI 201991 | *Secale* | *cereale* | *cereale* | P12 | 1.00 |
| SD_Sc019 | Pakistan | PI 218110 | *Secale* | *cereale* | *cereale* | P1 | 1.00 |
| SD_Sc020 | Pakistan | PI 219740 | *Secale* | *cereale* | *cereale* | P1 | 1.00 |
| SD_Sc021 | Pakistan | PI 219741 | *Secale* | *cereale* | *cereale* | P1 | 1.00 |
| SD_Sc022 | Afghanistan | PI 223896 | *Secale* | *cereale* | *cereale* | P1 | 1.00 |
| SD_Sc023 | Iran | PI 227870 | *Secale* | *cereale* | *cereale* | P12 | 1.00 |
| SD_Sc024 | Kazakhstan | PI 234655 | *Secale* | *cereale* | *cereale* | P1 | 1.00 |
| SD_Sc025 | Kazakhstan | PI 234656 | *Secale* | *cereale* | *cereale* | P12 | 1.00 |
| SD_Sc027 | France | PI 235536 | *Secale* | *cereale* | *cereale* | P2 | 2.67 |
| SD_Sc028 | Brazil | PI 239580 | *Secale* | *cereale* | *cereale* | P1 | 2.83 |
| SD_Sc029 | Argentina | PI 240676 | *Secale* | *cereale* | *cereale* | P1 | 1.00 |
| SD_Sc030 | Brazil | PI 241578 | *Secale* | *cereale* | *cereale* | P1 | 2.67 |
| SD_Sc032 | Iran | PI 243741 | *Secale* | *cereale* | *cereale* | P1 | 1.00 |
| SD_Sc033 | Greece | PI 249936 | *Secale* | *cereale* | *cereale* | P2 | 2.60 |
| SD_Sc034 | Iran | PI 250744 | *Secale* | *cereale* | *cereale* | P1 | 3.80 |
| SD_Sc039 | Austria | PI 254810 | *Secale* | *cereale* | *cereale* | P1 | 3.80 |
| SD_Sc040 | Spain | PI 256026 | *Secale* | *cereale* | *cereale* | P2 | 3.83 |
| SD_Sc041 | Switzerland | PI 263561 | *Secale* | *cereale* | *cereale* | P12 | 1.00 |
| SD_Sc042 | Estonia | PI 265471 | *Secale* | *cereale* | *cereale* | P1 | 1.00 |
| SD_Sc043 | Finland | PI 265473 | *Secale* | *cereale* | *cereale* | P12 | 1.00 |
| SD_Sc044 | Turkey | PI 266975 | *Secale* | *cereale* | *cereale* | P12 | 1.00 |
| SD_Sc045 | Latvia | PI 267098 | *Secale* | *cereale* | *cereale* | P12 | 1.00 |
| SD_Sc049 | Hungary | PI 272333 | *Secale* | *cereale* | *cereale* | P1 | 1.00 |
| SD_Sc050 | Afghanistan | PI 275356 | *Secale* | *cereale* | *cereale* | P1 | 2.83 |
| SD_Sc052 | Russian Federation | PI 280838 | *Secale* | *cereale* | *cereale* | P12 | 1.00 |
| SD_Sc053 | Russian Federation | PI 280841 | *Secale* | *cereale* | *cereale* | P1 | 1.00 |
| SD_Sc055 | Iran | PI 289814 | *Secale* | *cereale* | *cereale* | P1 | 2.67 |
| SD_Sc056 | Pakistan | PI 289827 | *Secale* | *cereale* | *cereale* | P12 | 2.00 |
| SD_Sc057 | Slovakia | PI 290423 | *Secale* | *cereale* | *cereale* | P12 | 2.80 |
| SD_Sc058 | Netherlands | PI 290425 | *Secale* | *cereale* | *cereale* | P1 | 1.00 |
| SD_Sc060 | Germany | PI 290435 | *Secale* | *cereale* | *cereale* | P12 | 2.80 |
| SD_Sc061 | Hungary | PI 290436 | *Secale* | *cereale* | *cereale* | P12 | 3.00 |
| SD_Sc062 | Ukraine | PI 290439 | *Secale* | *cereale* | *cereale* | P12 | 4.67 |
| SD_Sc063 | Finland | PI 290440 | *Secale* | *cereale* | *cereale* | P12 | 3.80 |
| SD_Sc066 | Bulgaria | PI 294794 | *Secale* | *cereale* | *cereale* | P12 | 1.20 |
| SD_Sc067 | Bulgaria | PI 294795 | *Secale* | *cereale* | *cereale* | P12 | 4.00 |
| SD_Sc069 | Romania | PI 306487 | *Secale* | *cereale* | *cereale* | P12 | 1.20 |
| SD_Sc070 | Romania | PI 306495 | *Secale* | *cereale* | *cereale* | P12 | 3.00 |
| SD_Sc072 | Brazil | PI 314964 | *Secale* | *cereale* | *cereale* | P1 | 3.00 |
| SD_Sc073 | France | PI 315957 | *Secale* | *cereale* | *cereale* | P12 | 3.20 |
| SD_Sc074 | Netherlands | PI 315962 | *Secale* | *cereale* | *cereale* | P1 | 1.83 |
| SD_Sc075 | Canada | PI 323363 | *Secale* | *cereale* | *cereale* | P12 | 2.00 |
| SD_Sc077 | United States | PI 323377 | *Secale* | *cereale* | *cereale* | P2 | 1.67 |
| SD_Sc078 | Spain | PI 323383 | *Secale* | *cereale* | *cereale* | P1 | 1.33 |
| SD_Sc081 | Poland | PI 323449 | *Secale* | *cereale* | *cereale* | P2 | 1.67 |
| SD_Sc082 | Poland | PI 323454 | *Secale* | *cereale* | *cereale* | P2 | 2.00 |
| SD_Sc083 | Austria | PI 326407 | *Secale* | *cereale* | *cereale* | P2 | 4.00 |
| SD_Sc084 | South Africa | PI 330413 | *Secale* | *cereale* | *cereale* | P2 | 2.17 |
| SD_Sc087 | Germany | PI 330424 | *Secale* | *cereale* | *cereale* | P12 | 4.00 |
| SD_Sc089 | South Africa | PI 330431 | *Secale* | *cereale* | *cereale* | P12 | 2.40 |
| SD_Sc091 | Sweden | PI 330439 | *Secale* | *cereale* | *cereale* | P2 | 3.00 |
| SD_Sc093 | Netherlands | PI 330445 | *Secale* | *cereale* | *cereale* | P12 | 2.17 |
| SD_Sc094 | United Kingdom | PI 330526 | *Secale* | *cereale* | *cereale* | P2 | 1.33 |
| SD_Sc096 | Poland | PI 338383 | *Secale* | *cereale* | *cereale* | P2 | 1.00 |
| SD_Sc097 | Montenegro | PI 344980 | *Secale* | *cereale* | *cereale* | P1 | 3.00 |
| SD_Sc098 | Macedonia | PI 344991 | *Secale* | *cereale* | *cereale* | P1 | 3.17 |
| SD_Sc099 | Macedonia | PI 344998 | *Secale* | *cereale* | *cereale* | P1 | 1.20 |
| SD_Sc100 | Serbia | PI 345000 | *Secale* | *cereale* | *cereale* | P1 | 2.17 |
| SD_Sc101 | United Kingdom | PI 345531 | *Secale* | *cereale* | *cereale* | P12 | 2.17 |
| SD_Sc102 | Australia | PI 345739 | *Secale* | *cereale* | *cereale* | P2 | 2.17 |
| SD_Sc103 | Australia | PI 345740 | *Secale* | *cereale* | *cereale* | P12 | 2.17 |
| SD_Sc104 | Australia | PI 346416 | *Secale* | *cereale* | *cereale* | P12 | 2.17 |
| SD_Sc107 | Montenegro | PI 349912 | *Secale* | *cereale* | *cereale* | P2 | 2.33 |
| SD_Sc109 | Bosnia and Herzegovina | PI 349923 | *Secale* | *cereale* | *cereale* | P12 | 2.17 |
| SD_Sc110 | Turkey | PI 357067 | *Secale* | *cereale* | *cereale* | P1 | 1.80 |
| SD_Sc111 | Croatia | PI 362391 | *Secale* | *cereale* | *cereale* | P1 | 1.00 |
| SD_Sc116 | Afghanistan | PI 366503 | *Secale* | *cereale* | *cereale* | P2 | 3.00 |
| SD_Sc117 | Sweden | PI 368157 | *Secale* | *cereale* | *cereale* | P12 | 3.67 |
| SD_Sc118 | Estonia | PI 372114 | *Secale* | *cereale* | *cereale* | P2 | 4.67 |
| SD_Sc119 | Ukraine | PI 372115 | *Secale* | *cereale* | *cereale* | P12 | 2.67 |
| SD_Sc120 | Belarus | PI 372116 | *Secale* | *cereale* | *cereale* | P12 | 2.83 |
| SD_Sc122 | Belarus | PI 372119 | *Secale* | *cereale* | *cereale* | P2 | 3.67 |
| SD_Sc127 | Serbia | PI 378230 | *Secale* | *cereale* | *cereale* | P1 | 2.17 |
| SD_Sc128 | Serbia | PI 378231 | *Secale* | *cereale* | *cereale* | P1 | 2.00 |
| SD_Sc129 | Macedonia | PI 378233 | *Secale* | *cereale* | *cereale* | P1 | 1.33 |
| SD_Sc131 | Macedonia | PI 378239 | *Secale* | *cereale* | *cereale* | P12 | 1.00 |
| SD_Sc134 | Germany | PI 392069 | *Secale* | *cereale* | *cereale* | P12 | 4.00 |
| SD_Sc136 | Lithuania | PI 404227 | *Secale* | *cereale* | *cereale* | P2 | 2.17 |
| SD_Sc141 | United Kingdom | PI 414080 | *Secale* | *cereale* | *cereale* | P12 | 1.50 |
| SD_Sc146 | India | PI 430004 | *Secale* | *cereale* | *cereale* | P1 | 1.33 |
| SD_Sc147 | Chile | PI 436165 | *Secale* | *cereale* | *cereale* | P12 | 3.40 |
| SD_Sc148 | Chile | PI 436171 | *Secale* | *cereale* | *cereale* | P2 | 3.67 |
| SD_Sc150 | Chile | PI 436192 | *Secale* | *cereale* | *cereale* | P2 | 4.00 |
| SD_Sc152 | Israel | PI 445980 | *Secale* | *cereale* | *cereale* | P2 | 1.20 |
| SD_Sc154 | Canada | PI 445984 | *Secale* | *cereale* | *cereale* | P1 | 3.17 |
| SD_Sc157 | Canada | PI 445998 | *Secale* | *cereale* | *cereale* | P2 | 3.17 |
| SD_Sc161 | Japan | PI 446020 | *Secale* | *cereale* | *cereale* | P2 | 3.17 |
| SD_Sc162 | Mexico | PI 446058 | *Secale* | *cereale* | *cereale* | P12 | 1.40 |
| SD_Sc163 | Lithuania | PI 446123 | *Secale* | *cereale* | *cereale* | P1 | 4.67 |
| SD_Sc167 | Greece | PI 446151 | *Secale* | *cereale* | *cereale* | P2 | 2.00 |
| SD_Sc168 | Poland | PI 446177 | *Secale* | *cereale* | *cereale* | P12 | 2.17 |
| SD_Sc169 | Latvia | PI 446181 | *Secale* | *cereale* | *cereale* | P2 | 1.33 |
| SD_Sc170 | Portugal | PI 446195 | *Secale* | *cereale* | *cereale* | P2 | 3.00 |
| SD_Sc173 | Romania | PI 446245 | *Secale* | *cereale* | *cereale* | P2 | 2.83 |
| SD_Sc176 | Estonia | PI 446514 | *Secale* | *cereale* | *cereale* | P1 | 3.50 |
| SD_Sc177 | China | PI 447337 | *Secale* | *cereale* | *cereale* | P2 | 3.00 |
| SD_Sc178 | China | PI 452132 | *Secale* | *cereale* | *cereale* | P1 | 1.67 |
| SD_Sc179 | China | PI 452133 | *Secale* | *cereale* | *cereale* | P1 | 3.67 |
| SD_Sc180 | United States | PI 464583 | *Secale* | *cereale* | *cereale* | P1 | 2.17 |
| SD_Sc182 | United States | PI 491395 | *Secale* | *cereale* | *cereale* | P1 | 3.00 |
| SD_Sc185 | United States | PI 522185 | *Secale* | *cereale* | *cereale* | P2 | 1.00 |
| SD_Sc186 | Morocco | PI 525203 | *Secale* | *cereale* | *cereale* | P2 | 2.40 |
| SD_Sc187 | Morocco | PI 525205 | *Secale* | *cereale* | *cereale* | P1 | 3.67 |
| SD_Sc191 | Italy | PI 534929 | *Secale* | *cereale* | *cereale* | P1 | 2.67 |
| SD_Sc195 | Romania | PI 534943 | *Secale* | *cereale* | *cereale* | P12 | 3.00 |
| SD_Sc197 | Ukraine | PI 534948 | *Secale* | *cereale* | *cereale* | P2 | 2.17 |
| SD_Sc201 | United States | PI 534954 | *Secale* | *cereale* | *cereale* | P2 | 3.00 |
| SD_Sc202 | Czechoslovakia | PI 534956 | *Secale* | *cereale* | *cereale* | P12 | 1.50 |
| SD_Sc203 | Austria | PI 534960 | *Secale* | *cereale* | *cereale* | P2 | 3.33 |
| SD_Sc204 | United States | PI 534961 | *Secale* | *cereale* | *cereale* | P2 | 1.83 |
| SD_Sc205 | United States | PI 534962 | *Secale* | *cereale* | *cereale* | P1 | 1.67 |
| SD_Sc209 | Belgium | PI 534970 | *Secale* | *cereale* | *cereale* | P12 | 2.80 |
| SD_Sc210 | Argentina | PI 534987 | *Secale* | *cereale* | *cereale* | P2 | 1.00 |
| SD_Sc211 | Argentina | PI 534988 | *Secale* | *cereale* | *cereale* | P12 | 1.00 |
| SD_Sc214 | Kenya | PI 535006 | *Secale* | *cereale* | *cereale* | P12 | 1.83 |
| SD_Sc215 | Austria | PI 535007 | *Secale* | *cereale* | *cereale* | P2 | 1.00 |
| SD_Sc219 | Portugal | PI 535083 | *Secale* | *cereale* | *cereale* | P1 | 1.00 |
| SD_Sc220 | Portugal | PI 535094 | *Secale* | *cereale* | *cereale* | P1 | 2.75 |
| SD_Sc225 | France | PI 535144 | *Secale* | *cereale* | *cereale* | P1 | 1.17 |
| SD_Sc227 | United States | PI 535154 | *Secale* | *cereale* | *cereale* | P12 | 1.83 |
| SD_Sc229 | United States | PI 535159 | *Secale* | *cereale* | *cereale* | P1 | 1.50 |
| SD_Sc230 | Romania | PI 535163 | *Secale* | *cereale* | *cereale* | P12 | 1.83 |
| SD_Sc231 | Uruguay | PI 535174 | *Secale* | *cereale* | *cereale* | P2 | 2.00 |
| SD_Sc239 | Poland | PI 535192 | *Secale* | *cereale* | *cereale* | P12 | 2.83 |
| SD_Sc241 | United States | PI 535199 | *Secale* | *cereale* | *cereale* | P2 | 2.33 |
| SD_Sc242 | Mexico | PI 542467 | *Secale* | *cereale* | *cereale* | P12 | 1.33 |
| SD_Sc243 | United States | PI 542469 | *Secale* | *cereale* | *cereale* | P2 | 2.00 |
| SD_Sc244 | Brazil | PI 542470 | *Secale* | *cereale* | *cereale* | P2 | 1.00 |
| SD_Sc246 | Argentina | PI 543398 | *Secale* | *cereale* | *cereale* | P1 | 4.33 |
| SD_Sc247 | Turkey | PI 543408 | *Secale* | *cereale* | *cereale* | P1 | 1.83 |
| SD_Sc249 | Turkey | PI 543593 | *Secale* | *cereale* | *cereale* | P1 | 3.00 |
| SD_Sc251 | Turkey | PI 543664 | *Secale* | *cereale* | *cereale* | P2 | 2.50 |
| SD_Sc254 | United States | PI 543729 | *Secale* | *cereale* | *cereale* | P1 | 2.17 |
| SD_Sc256 | United States | PI 552973 | *Secale* | *cereale* | *cereale* | P1 | 2.20 |
| SD_Sc257 | United States | PI 559980 | *Secale* | *cereale* | *cereale* | P2 | 4.00 |
| SD_Sc258 | United States | PI 559981 | *Secale* | *cereale* | *cereale* | P12 | 3.40 |
| SD_Sc261 | Turkey | PI 560572 | *Secale* | *cereale* | *cereale* | P2 | 1.00 |
| SD_Sc265 | Sweden | PI 561674 | *Secale* | *cereale* | *cereale* | P1 | 3.00 |
| SD_Sc271 | Turkey | PI 568106 | *Secale* | *cereale* | *cereale* | P1 | 1.00 |
| SD_Sc278 | Pakistan | PI 578092 | *Secale* | *cereale* | *cereale* | P1 | 1.00 |
| SD_Sc281 | Canada | PI 590948 | *Secale* | *cereale* | *cereale* | P2 | 3.00 |
| SD_Sc293 | United States | PI 628642 | *Secale* | *cereale* | *cereale* | P2 | 1.67 |
| SD_Sc296 | Tajikistan | PI 639328 | *Secale* | *cereale* | *cereale* | P1 | 1.00 |
| SD_Sc297 | Tajikistan | PI 639336 | *Secale* | *cereale* | *cereale* | P1 | 1.00 |
| SD_Sc269 | Pakistan | PI 561809 | *Secale* | *cereale* | *cereale* | P2 | 1.33 |
| SD_Sc326 | Armenia | PI 618662 | *Secale* | *cereale* | *afghanicum* | P12 | 2.33 |
| SD_Sc010 | Japan | Cise 107 | *Secale* | *cereale* | *ancestrale* | P12 | - |
| SD_Sc324 | Soviet Union | PI 445975 | *Secale* | *cereale* | *ancestrale* | P2 | 3.00 |
| SD_Sc327 | Turkey | PI 618663 | *Secale* | *cereale* | *ancestrale* | P1 | 3.00 |
| SD_Sc329 | Turkey | PI 618669 | *Secale* | *cereale* | *tetraploidum* | P2 | 2.00 |
| SD_Sc314 | Italy | Cise 105 | *Secale* | *cereale* | *segetale* | P1 | - |
| SD_Sc328 | Russian Federation | PI 618668 | *Secale* | *cereale* | *dighoricum* | P1 | - |
| SD_Sc321 | Azerbaijan | PI 267102 | *Secale* | *cereale* | *segetale* | P1 | - |
| SD_Sc325 | Ukraine | PI 573647 | *Secale* | *cereale* | *tetraploidum* | P2 | - |
| SD_Sc332 | South Africa | PI 630963 | *Secale* | *strictum* | *africanum* | P13 | - |
| SD_Sc323 | United States | PI 445973 | *Secale* | *strictum* | *anatolicum* | P12 | 2.66 |
| SD_Sc333 | Poland | PI 630967 | *Secale* | *strictum* | *ciliatoglume* | P3 | 2.50 |
| SD_Sc315 | Armenia | PI 592292 | *Secale* | *strictum* | *kuprijanovii* | P13 | 3.00 |
| SD_Sc334 | Poland | PI 630971 | *Secale* | *strictum* | *strictum* | P13 | 1.50 |
| SD_Sc330 | Ukraine | PI 618674 | *Secale* | *sylvestre* | *­-* | P3 | 3.00 |
| SD_Sc331 | Bulgaria | PI 618675 | *Secale* | *sylvestre* | *-* | P3 | 2.50 |
| SD_Sc320 | Afghanistan | PI 253957 | *Secale* | *vavilovii* | *-* | P1 | - |
| SD_Sc322 | Hungary | PI 284842 | *Secale* | *vavilovii* | *-* | P1 | - |
