# Supplementary material for: Assessing the genetic diversity and characterizing genomic regions conferring Tan Spot resistance in cultivated rye
Source: PLoS One. 2019 Mar 28;14(3):e0214519. doi: 10.1371/journal.pone.0214519 (PMC6438500; doi:10.1371/journal.pone.0214519)
Supplement: S3 Table — (DOCX) [file pone.0214519.s009.docx]

**S3 Table.** Nucleotide sequence flanking the SNPs associated with tan spot (*PTR* race 5) resistance in the global collection of *Secale cereale* accessions.

| **SNP** | **Nucleotide Sequence** |
| --- | --- |
| *S2R_6856816* | AACCTCATTTCCACCTTCCAGTGTGCCCTACATGAGTACATATTTTTCACTTGAACAAGTTGGAAACCGACTTTTTTTACTTCTAAAGTTACCCTTTCA**[T/C]**GGGTTTTATGCAGGAATAGGTAGATGATATGACACCAAAGACAAATAAGGATATGCCCTGCACGTTATCATTTCCATAACTGTTTTATTAACACAATAAG |
| *S5R_16433036* | TCTTGTACATACATATCTAGCTAGTGACAATTATATTGGTAGGAGGAGTATGTTGTCTGCAGAATTTTCCATAAGAGCACATGACTGAAGAAGGTGGTGA**[A/T]**GCCGTCATATGCCCCGCCCAGTCTTACGTCCATTGGGGCAGAACAACATCGGGGCTTCCTCGAATCCATTACATTGGCTCCACTCATGGATTACGGCGTN |
